# Parkin Impairs Antiviral Immunity by Suppressing the Mitochondrial Reactive Oxygen Species-Nlrp3 Axis and Antiviral Inflammation

**DOI:** 10.1016/j.isci.2019.06.008

**Published:** 2019-06-11

**Authors:** Jian Li, Chunmei Ma, Fei Long, Dongxue Yang, Xue Liu, Yingchao Hu, Chunyan Wu, Bingwei Wang, Min Wang, Yunzi Chen, Genyan Liu, Paul N. Moynagh, Jiawei Zhou, Tao Peng, Shuo Yang

**Affiliations:** 1 Department of Immunology, Key Laboratory of Immunological Environment and Disease, State Key Laboratory of Reproductive Medicine, Nanjing Medical University, Nanjing, China; 2 Laboratory of Viral Immunology, State Key Laboratory of Respiratory Disease, Sino-French Hoffmann Institute, Guangzhou Medical University, Guangzhou, China; 3 Department of Pharmacology, Nanjing University of Chinese Medicine, Nanjing, China; 4 Maynooth University Human Health Research Institute, Department of Biology, National University of Ireland Maynooth, Maynooth, Ireland; 5 Department of Laboratory Medicine, the First Affiliated Hospital of Nanjing Medical University, Nanjing 210029, China; 6 Centre for Experimental Medicine, Queen's University Belfast, Belfast, UK; 7 Institute of Neuroscience, State Key Laboratory of Neuroscience, Shanghai Institutes for Biological Sciences, Chinese Academy of Sciences, Shanghai, China

**Keywords:** Biological Sciences, Biochemistry, Genetics, Molecular Biology, Immunology, Cell Biology

## Abstract

Although mitochondria are known to be involved in host defense against viral infection, the physiological role of mitophagy, a crucial mechanism for maintaining mitochondrial homeostasis, in antiviral immunity remains poorly defined. Here, we show that Parkin, a central player in mitophagy, has a vital function in regulating host antiviral responses. Parkin-knockout mice exhibit improved viral clearance and survival after viral infection. However, Parkin deficiency does not affect antiviral signaling and interferon production. Instead, Parkin deficiency augments innate antiviral inflammation by enhancing mitochondrial ROS (mtROS)-mediated NLRP3 inflammasome activation and promoting viral clearance. Loss of NLRP3 can reverse the enhanced antiviral responses in Parkin knockout mice. Furthermore, we find that Parkin expression is downregulated in peripheral blood mononuclear cells of patients infected with virus. Collectively, our results suggest that Parkin plays an important role in antiviral immunity by controlling mtROS-NLRP3 axis-mediated inflammation. These findings provide physiological insight of the importance of mitophagy in regulating host antiviral response.

## Highlights

• Loss of Parkin enhances viral clearance but does not affect type I IFN production
• Parkin deletion promotes antiviral inflammation *in vivo*
• Parkin deficiency enhances antiviral inflammation via the mtROS-NLRP3 axis
• The expression of Parkin is downregulated following viral infection

## Graphical Abstract

**Figure:**
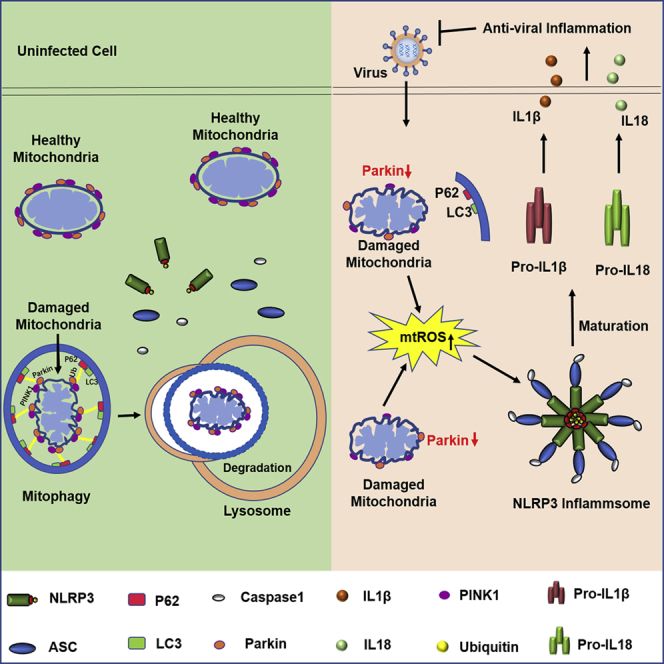

## Introduction

The innate immune response plays an essential role in defense against viral infection by the induction of antiviral cytokines and inflammation (Takeuchi and Akira, 2010). Host recognition of viral infection is mediated by different pattern-recognition receptors (PRRs), including Toll-like receptors (TLRs), RIG-I-like receptors (RLRs), and DNA sensors (Sun et al., 2010). Upon viral recognition, the PRRs trigger downstream signaling to activate transcription factors like nuclear factor (NF)-κB and IRFs and induce various inflammatory cytokines (Roers et al., 2016).

Mitochondria are the central hubs of cellular metabolism and have multifunctional roles in bioenergetic production, apoptosis, signal transduction, and the production of reactive oxygen species (ROS) (Chandel, 2014, Wallace and Fan, 2010). Mitochondria produce cellular ATP and metabolites, which are important in antiviral immune responses (Mills et al., 2017). Recent studies have shown that mitochondria participate in innate immune signaling to regulate antiviral responses (Weinberg et al., 2015, West et al., 2011). Mitochondria serve as a signaling platform in antiviral innate immunity. The mitochondrial membrane protein mitochondrial antiviral signaling protein (MAVS) is the central adaptor in RLR signaling and plays an important role in defense against RNA viruses (Kawai et al., 2005, Seth et al., 2005, Xu et al., 2005). NLRX1, another outer mitochondrial membrane (OMM) protein, functions as a negative regulator of inflammation by disrupting the MAVS and TRAF6-mediated pathways in response to viral infection (Allen et al., 2011, Moore et al., 2008). In addition, some published reports suggest that mitochondrial ROS (mtROS) can directly modulate antiviral signaling pathway, although its role remains controversial (Agod et al., 2017, Jin et al., 2010, Tal et al., 2009). Moreover, mtROS acts as an upstream signal to promote activation of the NLRP3 inflammasome (Zhong et al., 2018, Zhou et al., 2011). The NLRP3 inflammasome has been reported to drive antiviral inflammation and enhance the host immune response to viral infection (Allen et al., 2009, Ichinohe et al., 2009, Thomas et al., 2009, Wang et al., 2017).

Virus-induced mtROS is associated with mitochondrial dysfunction. Mitochondrial homeostasis is mediated by mitophagy, which decreases mtROS production by removing impaired mitochondria (Kubli and Gustafsson, 2012, Tal et al., 2009). Parkin, a cytosolic E3 ubiquitin ligase protein linked with Parkinson disease (PD), mediates mitophagy in conjunction with the ubiquitin kinase Pink1 that, like Parkin, is also a mutated gene in PD (Matheoud et al., 2016). Pink1 accumulates on the OMM when mitochondria are damaged or depolarized and assists the recruitment of Parkin to mitochondria where it ubiquitinates multiple mitochondrial proteins such as VDAC and TOMs. Ubiquitinated OMM proteins containing K63-linked polyubiquitin chains are recognized by the ubiquitin-binding domain of the specific autophagy receptor P62 that also has an LC3-binding domain that recruits LC3 and triggers mitophagy (Jin and Youle, 2012, Narendra et al., 2008). Thus the Pink1-Parkin pathway plays a key role in initiating mitophagy and regulating mitochondrial homeostasis. A recent study found that hepatitis B virus (HBV) can induce mitochondrial translocation of Parkin and subsequent mitophagy in hepatocytes (Kim et al., 2013). The same group further showed that during HBV infection the mitochondrially localized Parkin recruits Linear Ubiquitin Assembly Complex (LUBAC) to the mitochondria, leading to M-1 linked linear ubiquitination of MAVS and blockade of MAVS-mediated antiviral signaling (Khan et al., 2016). Moreover, during the preparation of this manuscript, another study reported that Parkin does not target MAVS but promotes K48 linkage ubiquitination and degradation of TRAF3 to suppress downstream antiviral signaling (Xin et al., 2018). However, these studies used a combination of overexpression and cell-line-based approaches, and the physiological role and mechanism of action of the mitophagy protein Parkin in antiviral response remains to be delineated. We now provide insight into the physiological function of Parkin in the host defense against viral infection while also describing the regulatory role of Parkin-dependent mitophagy in antiviral immunity.

Here we directly investigate the role of Parkin in host antiviral responses by using Parkin knockout mice (*Park2*^−/−^). We show that Parkin deficiency promotes viral clearance and mice survival after infection with both RNA and DNA viruses. Interestingly, Parkin does not affect antiviral signaling and interferon (IFN) production. Instead we discover that Parkin deficiency increases the innate inflammatory response to viral infection by augmenting mtROS-mediated NLRP3 activation. Moreover, we find that Parkin expression is downregulated in human peripheral blood mononuclear cells (PBMCs) during viral infection. These findings clearly demonstrate the importance of tight regulation of Parkin to maximize antiviral immunity.

## Results

### Loss of Parkin Enhances Viral Clearance and Mice Survival but Does Not Affect Type I IFN Production

**Figure 1:**
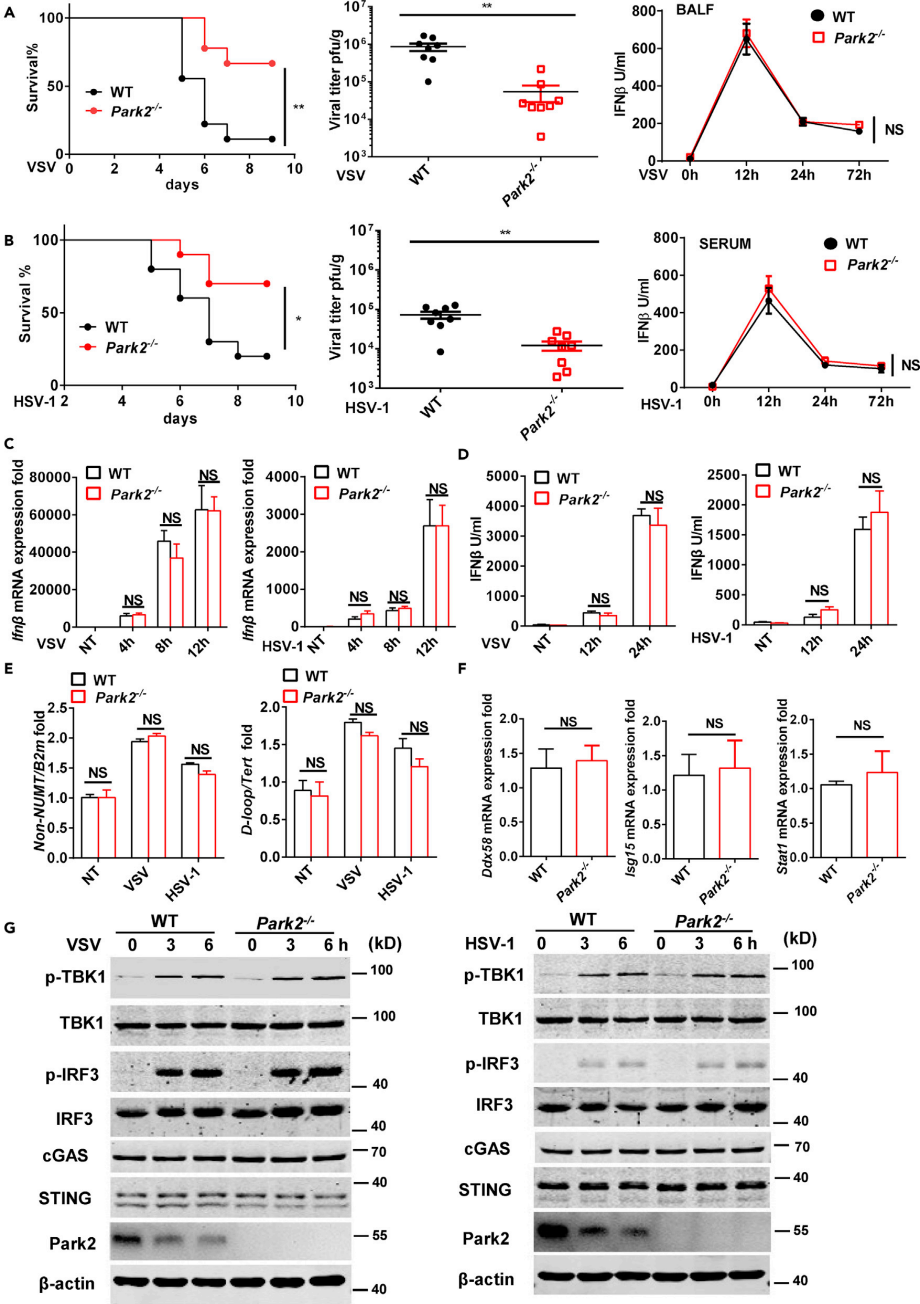
Parkin Deficiency Promotes Viral Clearance without Interfering with IFN Production (A) WT and *Park2*^*−/−*^ mice (n = 9) were intranasally infected with VSV at 2 × 10^8^ plaque-forming unit (PFU) per mouse, and the survival rates of mice were observed and recorded for each day post-infection. Viral titers of lung were determined by standard plaque assay 5 days after infection (n = 8). ELISA analysis of IFN-β of BALF from WT and *Park2*^*−/−*^ mice infected with VSV for 12, 24, and 72 h (n = 7 for viral infection, n = 3 for mock infection). (B) WT and *Park2*^*−/−*^ mice (n = 10) were intravenously infected with HSV-1 at 6 × 10^7^ PFU per mouse, and the survival rates of mice were observed and recorded for each day post-infection. Viral titers of lung were determined by standard plaque assay 5 days after infection (n = 8). ELISA analysis of IFN-β of serum from WT and Park2^−/−^ mice infected with HSV-1 for 12, 24, and 72 h (n = 7 for viral infection, n = 3 for mock infection). (C) RT-qPCR analysis of Ifnb mRNA expression of WT and *Park2*^*−/−*^ MEFs infected with VSV or HSV-1 at multiplicity of infection (MOI) 1 for 4, 8, and 12 h (n = 3).NT, no treatment. (D) ELISA analysis of IFN-β in the supernatants of WT and *Park2*^*−/−*^ MEFs infected with VSV or HSV-1 at MOI 1 for 12 and 24 h (n = 3). No treatment. (E) The mtDNA amounts were quantified by RT-qPCR with primers specific for the mitochondrial D loop region or a region of mtDNA that is not inserted into nuclear DNA (non-NUMT) and primers specific for nDNA (Tert, B2m) in WT and *Park2*^*−/−*^ MEFs infected with VSV or HSV-1 at MOI 10 for 4 h. NT, no treatment. (F) RT-qPCR analysis of *Ddx58*, *Isg15,* and *Stat1* mRNA expression of WT and *Park2*^*−/−*^ MEFs. (G) Immunoblot analysis of phosphorylated (p-) and total IRF3, phosphorylated (p-) TBK1 and total TBK1, cGAS, STING, Park2, and β-actin (loading control) in WT and *Park2*^*−/−*^ MEFs infected for 0, 3 and 6 h (above lanes) with VSV or HSV-1 at MOI 5. Data are pooled from three independent experiments. Error bars show means ± SEM. *p < 0.05, **p < 0.01, NS, not significant. Log rank (Mantel-Cox) test for survival rates, unpaired t test for viral titers, and two-way ANOVA with Sidak's multiple comparisons test for RT-qPCR and ELISA analyses. Related to Figures S1, S2, S6, and S8.

To study the physiological role of Parkin in viral infection, we utilized Parkin gene deletion mice (*Park2*^*−/−*^) for viral infection studies. First, we intranasally infected mice with vesicular stomatitis virus (VSV), a single-stranded RNA virus that activates RIG-I-MAVS antiviral signaling. We found that mice lacking Parkin exhibited significantly increased survival through 9 days post infection (dpi) and had lower viral loads in lung than wild-type (WT) mice after infection (Figure 1A), which is consistent with a previous report that Parkin functions as a negative regulator of MAVS antiviral signaling (Khan et al., 2016). Next, we intravenously infected mice with herpes simplex virus 1 (HSV-1, a DNA virus) to investigate if Parkin has a role in the response to DNA virus infection. We found that Parkin deficiency in mice also enhanced mice survival and reduced viral loads after viral infection compared with WT mice (Figure 1B). Given HSV-1 infection-induced innate antiviral response is independent of MAVS signaling (Paludan et al., 2011), these data suggest that Parkin can play a negative regulatory role in host defense against both RNA and DNA viruses, and we next addressed the mechanisms underlying these regulatory roles.

Type-1 IFNs play crucial roles in innate antiviral immunity by inducing the expression of hundreds of antiviral genes (Takeuchi and Akira, 2010). Because *Park2*^*−/−*^ mice exhibit a significant increase in viral clearance *in vivo*, we next sought to assess the effects of Parkin deficiency on IFN-β production. Unexpectedly, no significant differences in IFN-β production from bronchoalveolar lavage fluid (BALF), sera, and lung homogenates were detected between WT and *Park2*^*−/−*^ mice at early time points, such as 12, 24, and 72 h after VSV or HSV-1 infection (Figures 1A, 1B, and S1A), which is in contrast with the previously reported role of Parkin as a negative modulator of IFN production in cells (Xin et al., 2018). To further investigate any direct effect of Parkin on IFN-β production, we examined IFN expression in *ex vivo Park2*^*−/−*^ mouse embryonic fibroblasts (MEFs) challenged with VSV and HSV-1. Parkin-deficient MEFs exhibited similar expression levels of *Ifn* transcripts and IFN-β protein as WT MEFs in response to infection with VSV or HSV-1 (Figures 1C and 1D). Previous study showed that *Park2*^*−/−*^ mice following exhaustive exercise exhibits increased mitochondrial DNA (mtDNA) release from damaged mitochondria into cytosol, leading to activation of cGAS-STING-mediated IFN signaling (Sliter et al., 2018). We thus next investigate the abundance of mtDNA between WT and *Park2*^*−/−*^ MEFs. The mtDNA copy number was enhanced after viral infection, whereas the amount of mtDNA and expression of IFN-stimulated genes was not affected by Parkin deficiency (Figures 1E and 1F). Moreover, we evaluated early antiviral innate signaling after viral stimulation and observed no obvious differences in virus-induced activation of TBK1 or IRF3 and the protein levels of cGAS and STING between WT and *Park2*^*−/−*^ MEFs (Figure 1G). In addition, flow cytometry analysis showed that WT and *Park2*^*−/−*^ cells had similar susceptibility to VSV-GFP and HSV-1-GFP viral infection (Figure S1B) at various time points, which further suggests that Parkin does not affect the susceptibility of cells to initial viral infection or their ability to respond to viral infection by production of type I IFN. To further investigate any effect of Parkin on IFN-β production in response to non-replicative IFN inducer such as RLR ligands, we examined it by using poly(I:C) transfection and found that Parkin deficiency does not affect IFN production in response to this MAVS-dependent PAMP (Figures S8C and S8D). To address potential cell type differences, we also assessed the effect of Parkin deficiency in bone marrow-derived dendritic cells (BMDCs) and bone marrow-derived macrophage cells (BMDMs). Like MEFs, there were no differences in virus-induced IFN production and IRF3-dependent signaling between WT and *Park2*^*−/−*^ BMDCs or BMDMs (Figures S2A–S2E). Collectively, these results clearly indicate that Parkin has no direct influence on innate antiviral signaling that triggers type I IFN production.

### Parkin Deletion Promotes Antiviral Inflammation *In Vivo*

**Figure 2:**
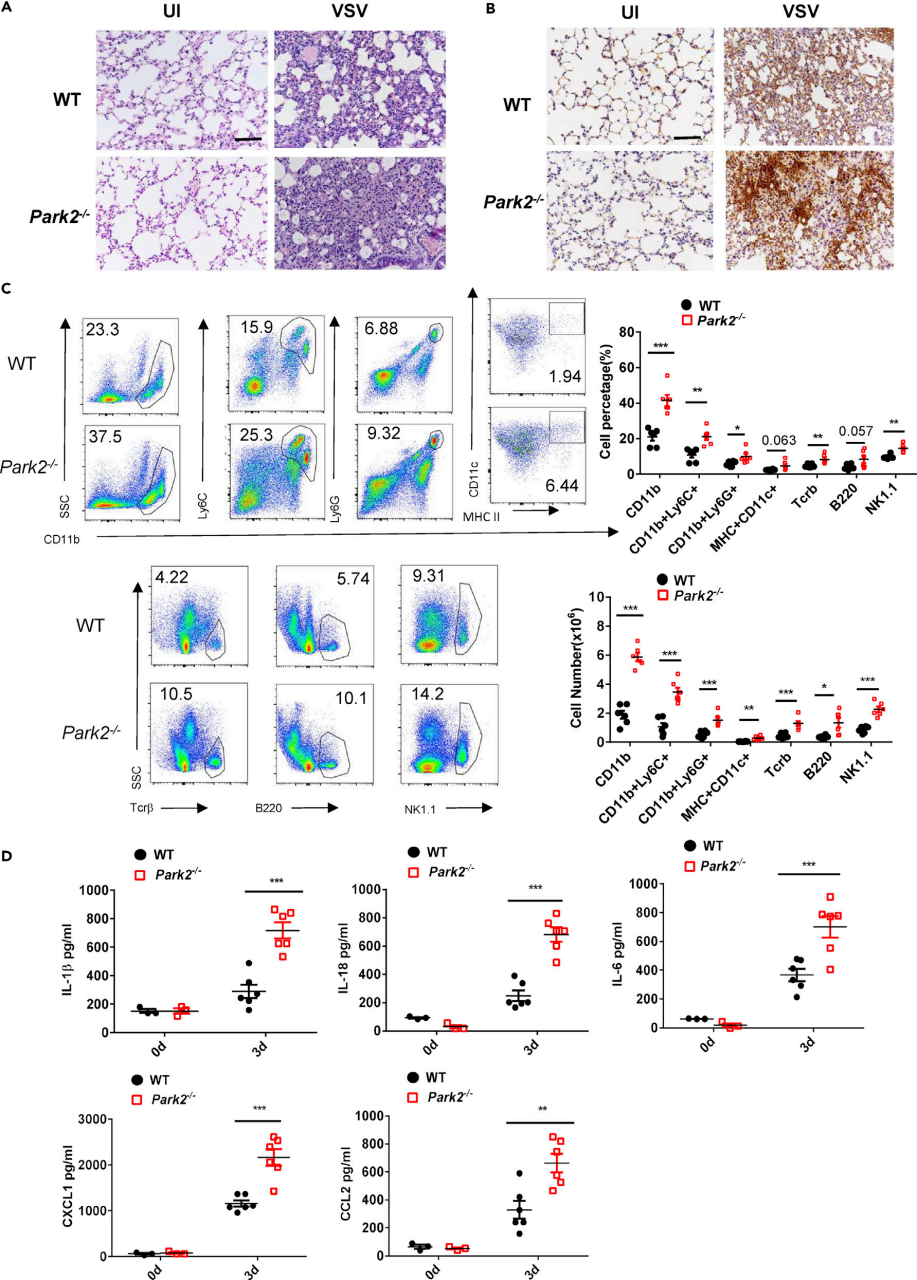
Parkin-Deficient Mice Exhibit an Enhanced Antiviral Inflammatory Response to VSV Infection (A) Hematoxylin and eosin staining of lung sections from WT and *Park2*^*−/−*^ mice 3 days after infection with VSV at 2 × 10^8^ plaque-forming unit per mouse (n = 3). Scale bar, 100 μm. (B) Immunohistochemical detection of myeloid cells (CD11b^+^) in lungs of WT and *Park2*^*−/−*^ mice infected as indicated in (A) (n = 3). Scale bar, 100 μm. (C) Flow cytometry analysis of the numbers of myeloid cells (CD11b^+^), inflammatory monocytes (CD11b^+^Ly6C^+^), neutrophils (CD11b^+^Ly6G^+^), dendritic cells (CD11b^+^/CD11c^+^/MHC II^+^), T cells (Tcr β^+^), B cells (B220^+^), and NK cells (NK1.1^+^) in lungs of WT and Park2^−/−^ mice infected as indicated in (A) (n = 6 mice per group). (D) ELISA analysis of IL-1β, IL-18, IL-6, CXCL1, and CCL2 of BALF from WT and *Park2*^*−/−*^ mice infected as indicated in (A) (n = 6 for viral infection, n = 3 for mock infection). Data are pooled from three independent experiments. Error bars show means ± SEM. *p < 0.05, **p < 0.01, ***p < 0.001. Two-way ANOVA with Sidak's multiple comparisons test for ELISA. Related to Figures S3 and S4.

**Figure 3:**
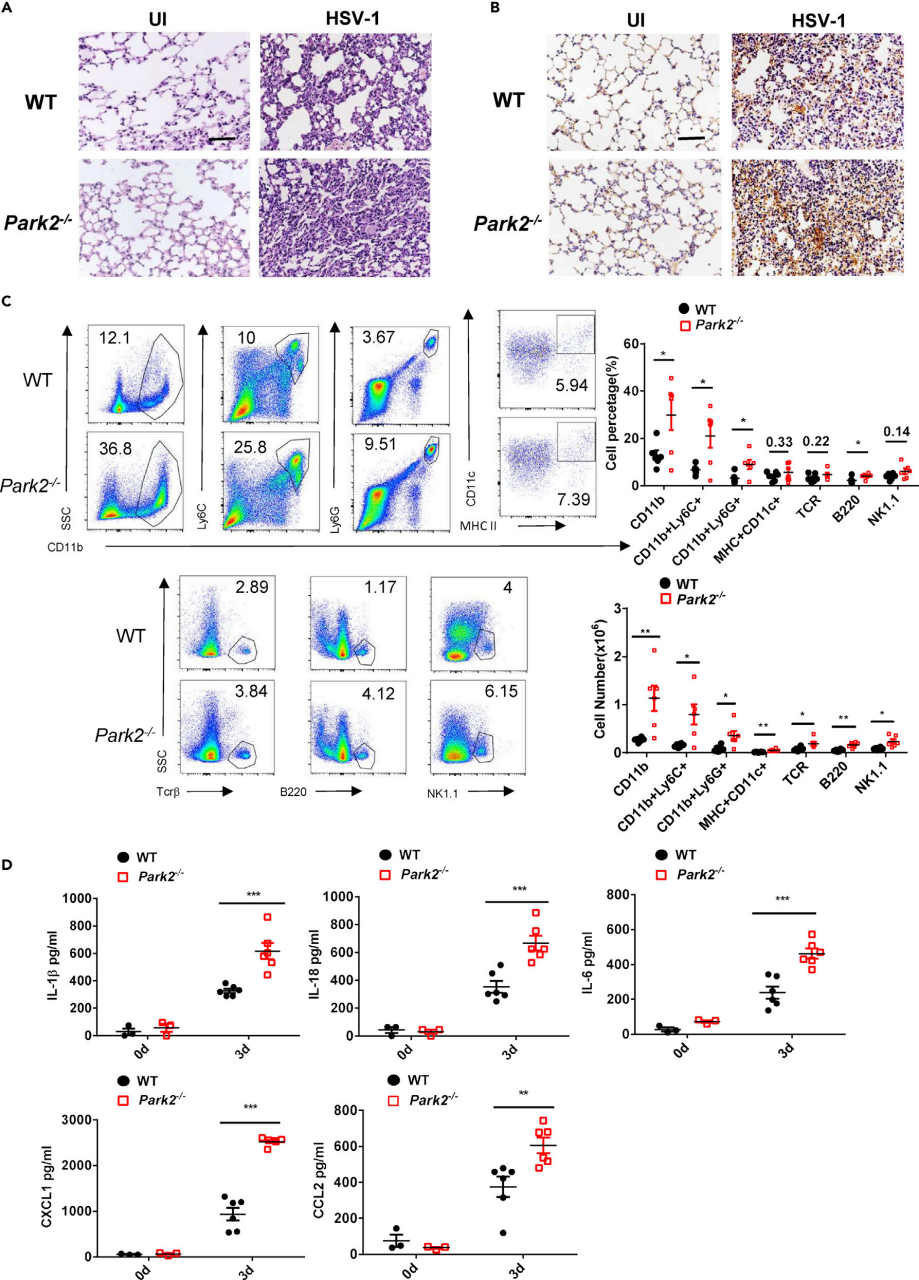
Parkin-Deficient Mice Exhibit an Enhanced Antiviral Inflammatory Response to HSV-1 Infection (A) Hematoxylin and eosin staining of lung sections from WT and *Park2*^*−/−*^ mice 3 days after intravenous injection of HSV-1 (6 × 10^7^ plaque-forming unit per mouse) (n = 3). Scale bar, 100 μm. (B) Immunohistochemical detection of myeloid cells (CD11b^+^) in lungs of WT and *Park2*^*−/−*^ mice infected as indicated in (A) (n = 3). Scale bar, 100 μm. (C) Flow cytometry analysis and numbers of myeloid cells (CD11b^+^), inflammatory monocytes (CD11b^+^Ly6C^+^), neutrophils (CD11b^+^Ly6G^+^), dendritic cells (CD11b^+^/CD11c^+^/MHC II^+^), T cells (Tcrβ^+^), B cells (B220^+^), and NK cells (NK1.1^+^) in lungs of WT and *Park2*^*−/−*^ mice infected as indicated in (A) (n = 6 mice per group). (D) ELISA analysis of IL-1β, IL-18, IL-6, CXCL1, and CCL2 of serum from WT and *Park2*^*−/−*^ mice infected as indicated in (A) (n = 6 for viral infection, n = 3 for mock infection). Data are pooled from three independent experiments. Error bars show means ±SEM. *p < 0.05, **p < 0.01, ***p < 0.001. One unpaired t test with Holm-Sidak method for FACS and two-way ANOVA with Sidak's multiple comparisons test for ELISA. Related to Figures S3 and S4.

During viral infection, the initial inflammatory response can contribute to viral clearance by recruiting and activating immune cells to combat viral invasion (Chen et al., 2011, Rouse and Sehrawat, 2010). Thus, we next assessed if Parkin can affect antiviral inflammation in an *in vivo* setting. Histological analysis revealed that lungs in *Park2*^*−/−*^ mice contained more severe inflammation characterized by more infiltration of immune cells than lungs from WT mice after the early stage of VSV infection (at 3 dpi) (Figure 2A). This was further verified by immunohistochemical detection of increased infiltration of CD11b^+^ myeloid cells in lungs from *Park2*^*−/−*^ mice (Figure 2B). Moreover, fluorescence-activated cell sorting (FACS) analysis showed that *Park2*^*−/−*^ mice had markedly increased percentages and numbers of CD11b^+^ cells in lungs on day 3 after VSV infection compared with WT mice (Figure 2C). In relation to CD11b^+^ subsets of cells, inflammatory monocytes (Ly6C^+^) and neutrophils (Ly6G^+^) were significantly increased in *Park2*^*−/−*^ mice. In addition, there were increases in the numbers of monocyte-macrophage DCs (dendritic cells) (CD11b^+^/CD11c+/MHC II^+^), NK cells (NK1.1), T (Tcrβ^+^), and B (B220^+^) lymphatic cells in lungs of *Park2*^*−/−*^ mice (Figure 2C). Consistent with these results, we found that the production of inflammatory cytokines and chemokines, including

interleukin (IL)-1β, IL-18, IL-6, CXCL1, and CCL2, were significantly increased in BALF (Figure 2D) and lung (Figure S3A) of *Park2*^*−/−*^ mice on 3 dpi VSV compared with WTs. These data suggest that Parkin is important for regulating antiviral inflammation during VSV infection. To determine whether Parkin is involved in the antiviral inflammatory response against DNA viruses, we next performed histological, immunohistochemical, FACS and ELISA analyses in *Park2*^*−/−*^ mice challenged with HSV-1 virus. Like VSV infection, the lung inflammatory histopathology (Figure 3A); the infiltration of immune cells into lung, as evidenced by immunohistochemistry (Figure 3B) and FACS (Figure 3C); and the levels of inflammatory cytokines in sera (Figure 3D) or lung (Figure S3B) were all markedly increased in *Park2*^*−/−*^ mice on 3 dpi HSV-1 compared with WT controls. Altogether, these findings clearly demonstrate that Parkin deletion augments the general antiviral inflammatory response, which contributes to enhanced viral clearance.

### Parkin Deficiency Enhances the Production of Inflammasome-Related Cytokines after Viral Infection

**Figure 4:**
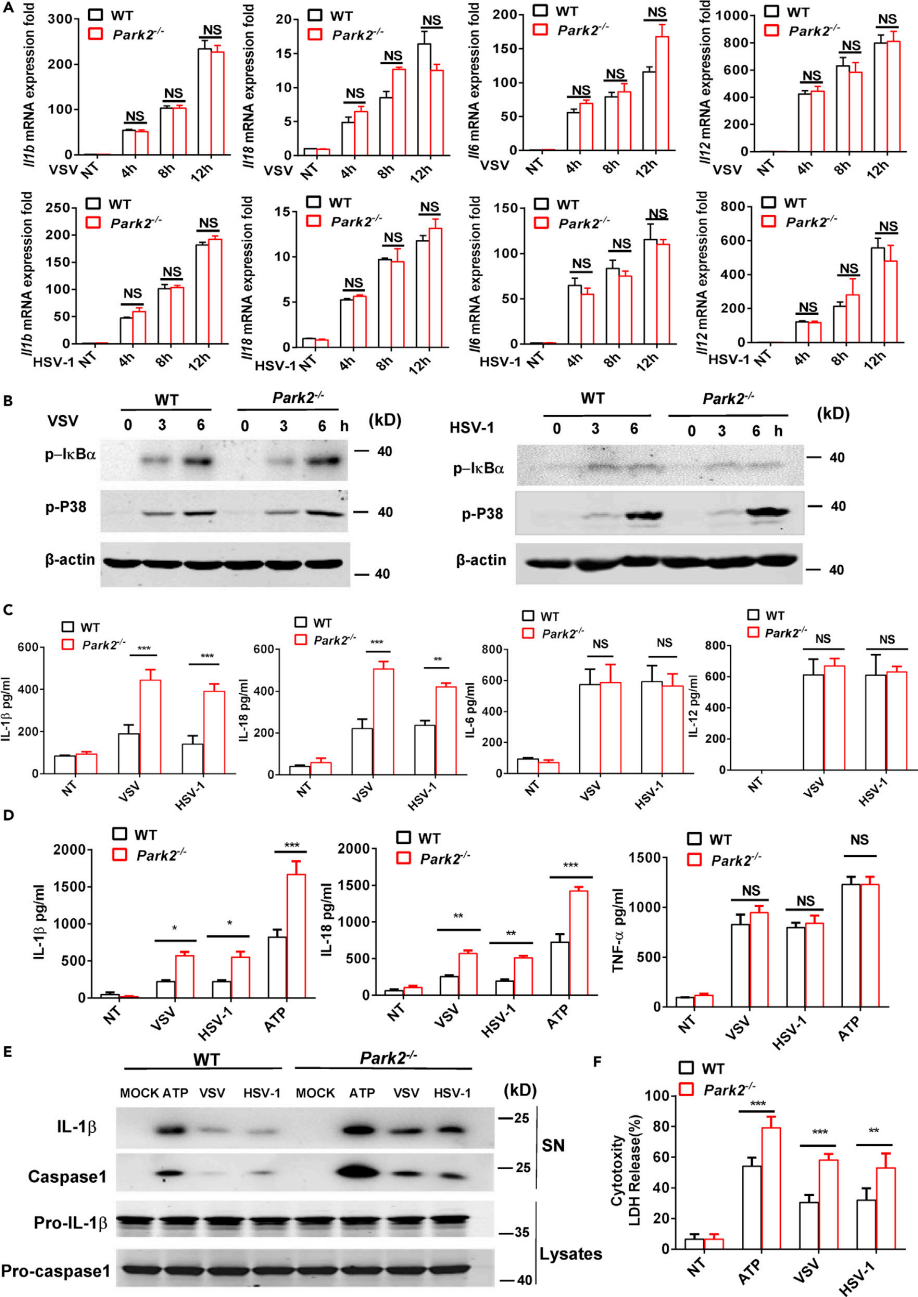
Parkin Deficiency in Myeloid Linage Cells Increases the Production of Inflammasome-Related Cytokines in Response to Viral Infection (A) RT-qPCR analysis of *Il1b*, *Il18*, *Il6,* and *Il12* mRNA expression of WT and *Park2*^*−/−*^ BMDCs infected with VSV or HSV-1 at MOI 1 for 4, 8, and 12 h. NT, no treatment. (B) Immunoblot analysis of phosphorylated (p-) IκBα, phosphorylated (p-) P38, and β-actin (loading control) in WT and *Park2*^*−/−*^ BMDCs infected for 0, 3, and 6 h with VSV or HSV-1 at MOI 5. (C) ELISA analysis of IL-1β, IL-18, IL-6, and IL-12 in the supernatants of WT and *Park2*^*−/−*^ BMDCs infected with VSV at MOI 1 or HSV-1 at MOI 2 for 24 h. NT, no treatment. (D) ELISA analysis of IL-1β, IL-18, and TNF-α in the supernatants of Pam3CSK4-primed WT and *Park2*^*−/−*^ BMDMs treated with ATP for 1 h and infected with VSV at MOI 1 or HSV-1 at MOI 2 for 24 h. NT, no treatment. (E) Immunoblot analysis of cleaved IL-1β and caspase-1 in culture supernatants (SN) of Pam3CSK4-primed WT and *Park2*^*−/−*^ BMDMs stimulated with VSV at MOI 5 or HSV-1 at MOI 10 for 24 h and immunoblot analysis of the precursors of IL-1β (Pro-IL-1β) and caspase-1 (Pro-caspase1) in lysates of those cells (lysates). (F) LDH (lactate dehydrogenase) assay of Pam3CSK4-primed WT and *Park2*^*−/−*^ BMDMs stimulated as indicated in (D). NT, no treatment. Data are pooled from three independent experiments (A, C, D, and F) or are representative of two independent experiments (B and E). Error bars show means ± SEM. *p < 0.05, **p < 0.01, ***p < 0.001, NS, not significant. two-way ANOVA with Sidak's multiple comparisons test. Related to Figure S5.

Although adaptive immunity can contribute to the antiviral inflammatory response (Dorner and Radbruch, 2007, Swain et al., 2012), Parkin deficiency had no effects on the profiles of CD4^+^, CD8^+^, CD44^+^, CD62L^+^ T (Figures S4A, S4C, and S4D), and CD19^+^ B (Figure S4B) lymphatic cells in thymus and spleen, suggesting that the enhanced antiviral inflammation in *Park2*^*−/−*^ mice is not associated with differences in cells of the adaptive immune system. Given our earlier data above demonstrating that CD11b^+^ myeloid cells represented the bulk of the lung-infiltrating cell population in *Park2*^*−/−*^ mice at day 3 after viral infection (Figures 2C and 3C) and the essential role of myeloid lineage cells in initiating antiviral inflammation (Varol et al., 2015), we next investigated the effect of Parkin on the production of virus-induced inflammatory cytokines in BMDCs. VSV and HSV-1 induced expression of mRNA transcripts of cytokines, such as *Il1b*, *Il18*, *Il6,* and *Il12*, were largely comparable between WT and *Park2*^*−/−*^ cells after viral infection (Figure 4A). In addition, early signaling pathways, such as activation of NF-κB and p38 mitogen-associated protein kinase, that mediate virus-induced transcription of these genes, were unaffected in Parkin-deficient cells as evidenced by intact VSV and HSV-1 induced phosphorylation of IκBα and p38 in *Park2*^*−/−*^ cells (Figure 4B). However, *Park2*^*−/−*^ BMDCs produced higher amounts of inflammasome-related cytokines, including IL-1β and IL-18, in response to VSV or HSV-1 infection, whereas there were no differences in the virus-induced production of other cytokines such as IL-6 and IL-12 between WT and *Park2*^*−/−*^ cells (Figure 4C). These findings suggest that Parkin in myeloid cells negatively regulates virus-induced production of inflammasome-related cytokines by targeting post-transcriptional regulation of their expression. To extrapolate these findings into a human setting, we further evaluated the effect of Parkin deficiency on the production of cytokines in the human monocyte cell line THP-1 after viral infection. THP-1 cells were transfected with small interfering RNA to knockdown Parkin expression (Figure S5A) and then infected with VSV or HSV-1. The production of IL-1β and IL-18 was significantly enhanced in Parkin knockdown cells as determined by ELISA assay, whereas virus-induced expression of other cytokines, such as IL-6 and IFN-β, was not affected (Figure S5B). Thus, these results confirm that the selective targeting in the production of inflammasome-related cytokines by Parkin applies to murine and human cells. Although Parkin deficiency does not affect viral induced expression of IL-6 in cell infection studies, it is interesting to note from our earlier data that *Park2*^*−/−*^ mice show augmented lung levels of IL-6 in response to viral infection. Such differences in cell- and animal-based infection approaches are reconciled by previous studies that have shown inflammasome-related cytokines, such as IL-1β and IL-18, to induce the production of proinflammatory cytokines and chemokines (Brabcova et al., 2014, Cahill and Rogers, 2008, McGeough et al., 2012, Yoo et al., 2005). Therefore the enhanced production of IL-6, CXCL1, and CCL2 in lungs of virus-infected *Park2*^*−/−*^ mice is a likely secondary consequence of the higher amounts of IL-1β and IL-18.

Given that Parkin does not appear to regulate the transcription of genes encoding IL-1β and IL-18, coupled to a recent report showing that Parkin inhibits ATP-induced NLRP3 inflammasome activation (Zhong et al., 2016), we next explored if Parkin can affect virus-induced inflammasome activation in BMDMs. To this end, Pam3CSK4 (TLR2 agonist)-primed BMDMs were challenged with VSV, HSV-1, and ATP as a second signal control for NLRP3 activation. ELISA analysis showed that IL-1β and IL-18 secretion was markedly augmented in *Park2*^*−/−*^ cells relative to WT counterparts after viral infection, whereas the production of tumor necrosis factor (TNF)-α, an inflammasome-independent cytokine, was not affected by *Park2* deletion (Figure 4D). This was further confirmed by western blot analysis showing that the release of mature IL-1β and caspase-1 in cell supernatants was significantly increased in virus-infected *Park2*^*−/−*^ cells (Figure 4E). Moreover, the deficiency of Parkin in BMDMs augmented virus-induced lactate dehydrogenase (LDH) release (Figure 4F), a measure of pyroptotic cell death that is also triggered by NLRP3 activation. Together, all these results indicate that Parkin in myeloid lineage cells has a unique role in inhibiting virus-induced inflammasome activation and subsequent production of IL-1β and IL-18 and pyroptosis.

### Parkin Restricts Mitochondrial Damage, mtROS Overproduction, and mtROS-Induced Inflammasome Activation during Viral Infection

**Figure 5:**
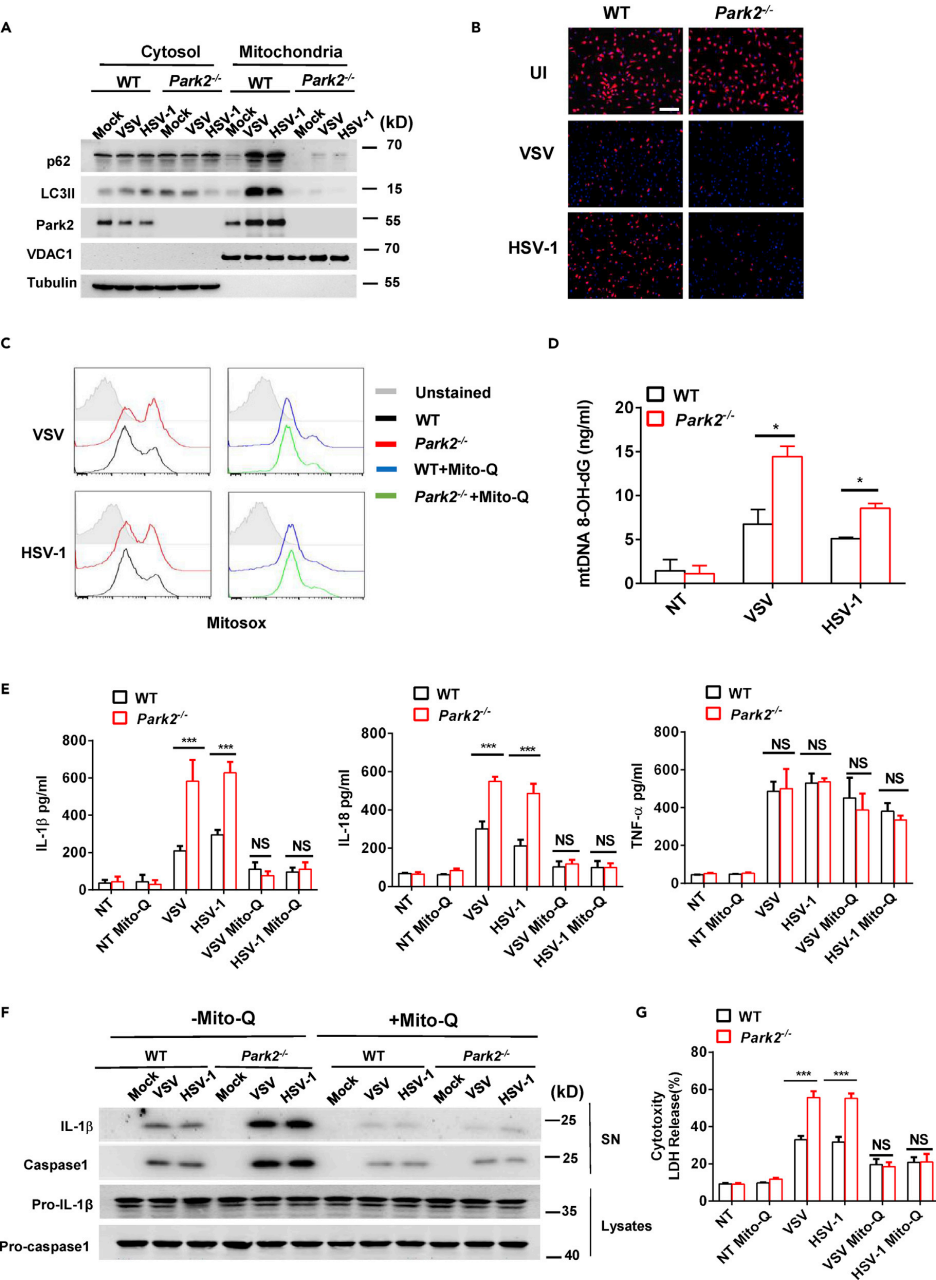
Parkin Restricts Mitochondrial Damage, mtROS Overproduction, and mtROS-Induced Inflammasome Activation during Viral Infection (A) Immunoblot analysis of p62, LC3II, and Park2 in cytosolic and mitochondrial subcellular fractions from Pam3CSK4-primed WT and *Park2*^*−/−*^ BMDMs infected with VSV at MOI 5 or HSV-1 at MOI 10 for 5 h. (B) Microscopic analysis of mitochondrial membrane potential of cells stimulated with VSV or HSV-1 at MOI 1 by TMRM (tetramethylrhodamine, methyl ester) fluorescence. UI, uninfected. Scale bar, 100 μm. (C) Flow cytometry analysis of the levels of mitochondria-associated ROS (mtROS) labeled by MitoSOX in Pam3CSK4-primed WT and *Park2*^*−/−*^ BMDMs untreated or treated with 50 nM Mito-Q (mitochondrial-specific antioxidant) followed by VSV or HSV-1 infection at MOI 5 for 12 h. (D) ELISA analysis of 8-OH-dG in the cytosol of Pam3CSK4-primed WT and *Park2*^*−/−*^ BMDMs treated with VSV and HSV-1 at MOI 10 for 5 h. (E) ELISA analysis of IL-1β, IL-18, and TNF-α in the supernatants of Pam3CSK4-primed WT and *Park2*^*−/−*^ BMDMs untreated or treated with 50 nM Mito-Q and then infected with VSV at MOI 1 or HSV-1 at MOI 2 for 24 h (n = 3). (F) Immunoblot analysis of cleaved IL-1β and caspase-1 in culture supernatants (SN) of Pam3CSK4-primed WT and *Park2*^*−/−*^ BMDMs treated with 50 nM Mito-Q and then infected with VSV at MOI 5 or HSV-1 at MOI 10 for 24 h. (G) LDH assay of Pam3CSK4-primed WT and *Park2*^*−/−*^ BMDMs treated as indicated in (E). Data are pooled from three (D, E, and G) independent experiments or are representative of two independent experiments (A, B, C, and F). Error bars show means ± SEM. *p < 0.05, ***p < 0.001. NS, not significant. Unpaired t test or two-way ANOVA with Sidak's multiple comparisons test.

We next probed the mechanism by which Parkin targets activation of the NLRP3 inflammasome. Mitochondrial dysfunction induces the production of mtROS that can trigger NLRP3 inflammasome activation (Zhou et al., 2011). As a central mitophagy regulator, Parkin is linked to the removal of damaged mitochondria (Youle and Narendra, 2011). We therefore examined whether Parkin could control virus-induced mitochondrial dysfunction and mtROS production in BMDMs. To investigate this, we first performed cell fractionation to isolate mitochondrial and cytosolic fractions and used western blot analysis to monitor recruitment of autophagy proteins such as P62 and lipidated LC3 (LC3II) to damaged mitochondria that are destined for clearance by mitophagy. Infection of WT BMDMs with VSV or HSV-1 showed mitochondrial enrichment of the autophagy proteins P62, LC3II, and Parkin, whereas the mitochondrial recruitment of these proteins was abolished in Parkin-deficient cells (Figure 5A), thus confirming the central role of Parkin in virus-induced mitophagy. Moreover, we found that loss of Parkin led to more severe virus-induced loss of mitochondrial membrane potential as indicated by reduced mitochondrial accumulation of the fluorescence dye TMRM (Figure 5B), indicating the increased mitochondrial damage in *Park2*^*−/−*^ cells. In addition, FACS analysis showed that viral infection induced more mtROS production in *Park2*^*−/−*^ than WT cells (Figure 5C). Consistent with this, the oxidized mtDNA (ox-mtDNA) significantly increased in the cytosol of *Park2*^*−/−*^ cells after viral infection (Figure 5D). These data strongly suggest that Parkin inhibits virus-induced mtROS production and ox-mtDNA release by restricting accumulation of damaged mitochondria.

ROS produced by damaged mitochondria is required for the production of ox-mtDNA, which is an important trigger of NLRP3 inflammasome activation (Yu et al., 2014, Zhong et al., 2018, Zhou et al., 2011). Thus, we reasoned that mtROS might be required for increased inflammasome activation in Parkin-deficient cells after viral infection. To test this hypothesis, we treated macrophages with the mitochondrial-specific antioxidant Mito-Q before viral challenge. Indeed, Mito-Q treatment blocked virus-induced mtROS production in WT and *Park2*^*−/−*^ cells (Figure 5C). Interestingly, the virus-induced secretions of IL-1β and IL-18 (Figure 5E) were also reduced by Mito-Q to basal levels in both WT and *Park2*^*−/−*^ BMDMs, whereas virus-induced expression of TNF was unaffected by Mito-Q (Figure 5E). The inhibitory effects of Mito-Q on inflammasome-mediated cytokine processing was also confirmed by western blot analysis that showed Mito-Q to block the release of mature IL-1β and caspase-1 in supernatants of virus-infected WT and *Park2*^*−/−*^ cells (Figure 5F). Mito-Q also blocked the ability of VSV and HSV-1 to promote cytotoxicity in both WT and *Park2*^*−/−*^ cells as demonstrated by greatly reduced release of LDH (Figure 5G). Collectively, these results indicate that mtROS is required for the enhanced activation of inflammasome in *Park2*^*−/−*^ cells after viral infection.

### Enhanced Antiviral Inflammation and Viral Clearance in Parkin-Deficient Mice Is Dependent on NLRP3 Inflammasome

**Figure 6:**
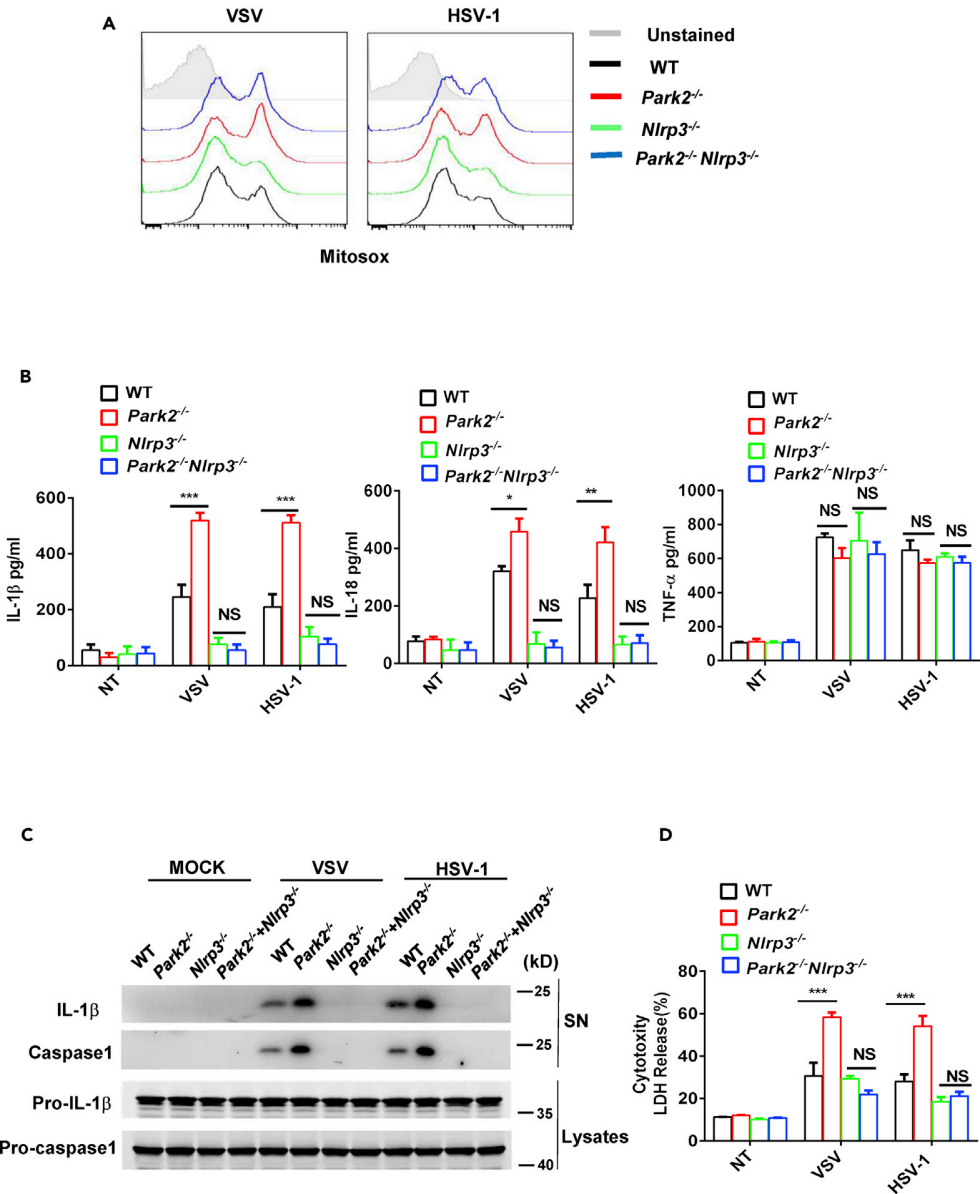
NLRP3 Deficiency Inhibits the Overproduction of Inflammasome-Related Cytokines in Parkin-Deficient Cells during Viral Infection (A) Flow cytometry analysis of mtROS in Pam3CSK4-primed WT, *Park2*^*−/−*^, *Nlrp3*^*−/−*^, and *Park2*^*−/−*^*Nlrp3*^*−/−*^ BMDMs infected with VSV or HSV-1 at MOI 5 for 12 h. (B) ELISA analysis of IL-1β, IL-18, and TNF-α in the supernatants of Pam3CSK4-primed WT, *Park2*^*−/−*^, *Nlrp3*^*−/−*^, and *Park2*^*−/−*^*Nlrp3*^*−/−*^ BMDMs infected with VSV at MOI 1 or HSV-1 at MOI 2 for 24 h. (C) Immunoblot analysis of cleaved IL-1β and caspase-1 in culture supernatants (SN) of Pam3CSK4-primed WT, *Park2*^*−/−*^, *Nlrp3*^*−/−*^, and *Park2*^*−/−*^*Nlrp3*^*−/−*^ BMDMs infected with VSV at MOI 5 or HSV-1 at MOI 10 for 24 h. (D) LDH assay of Pam3CSK4-primed WT, *Park2*^*−/−*^, *Nlrp3*^*−/−*^, and *Park2*^*−/−*^*Nlrp3*^*−/−*^ BMDMs infected as in (B). Data are pooled from three (B and D) independent experiments or are representative of two independent experiments (A and C). Error bars show means ± SEM. *p < 0.05, **p < 0.01, ***p < 0.001, NS, not significant. Two-way ANOVA with Tukey's multiple comparisons test. Related to Figure S7.

To further confirm if the effect of Parkin on virus-induced inflammasome activation is NLRP3 dependent, we crossed *Park2*^−/−^ and ^*-*^*Nlrp3*^*−/−*^ mice to make double knockout BMDMs and then characterized inflammasome activation in these cells. The absence of NLRP3 in *Park2*^−/−^ BMDMs had no effect on virus-induced mtROS production (Figure 6A), and this is hardly surprising because mtROS production is upstream of NLRP3. However, VSV or HSV-1-induced release of IL-1β and IL-18 was blocked in *Park2*^−/−^ *Nlrp3*^*−/−*^ double knockout cells with this suppressive effect being selective for inflammasome-related cytokines because virus-induced production of TNF was fully intact in double knockout cells (Figure 6B). This was further confirmed by western blot analysis showing that NLRP3 deficiency blocked the secretion of mature IL-1β and caspase-1 in supernatant of *Park2*^*−/−*^ cells after viral infection (Figure 6C). Furthermore, the absence of NLRP3 in double knockout cells also precluded the augmented virus-induced release of LDH that is normally observed in *Park2*^*−/−*^ cells (Figure 6D). All these findings are fully consistent with Parkin deficiency promoting virus-induced inflammasome activation via the mtROS-NLRP3 axis.

**Figure 7:**
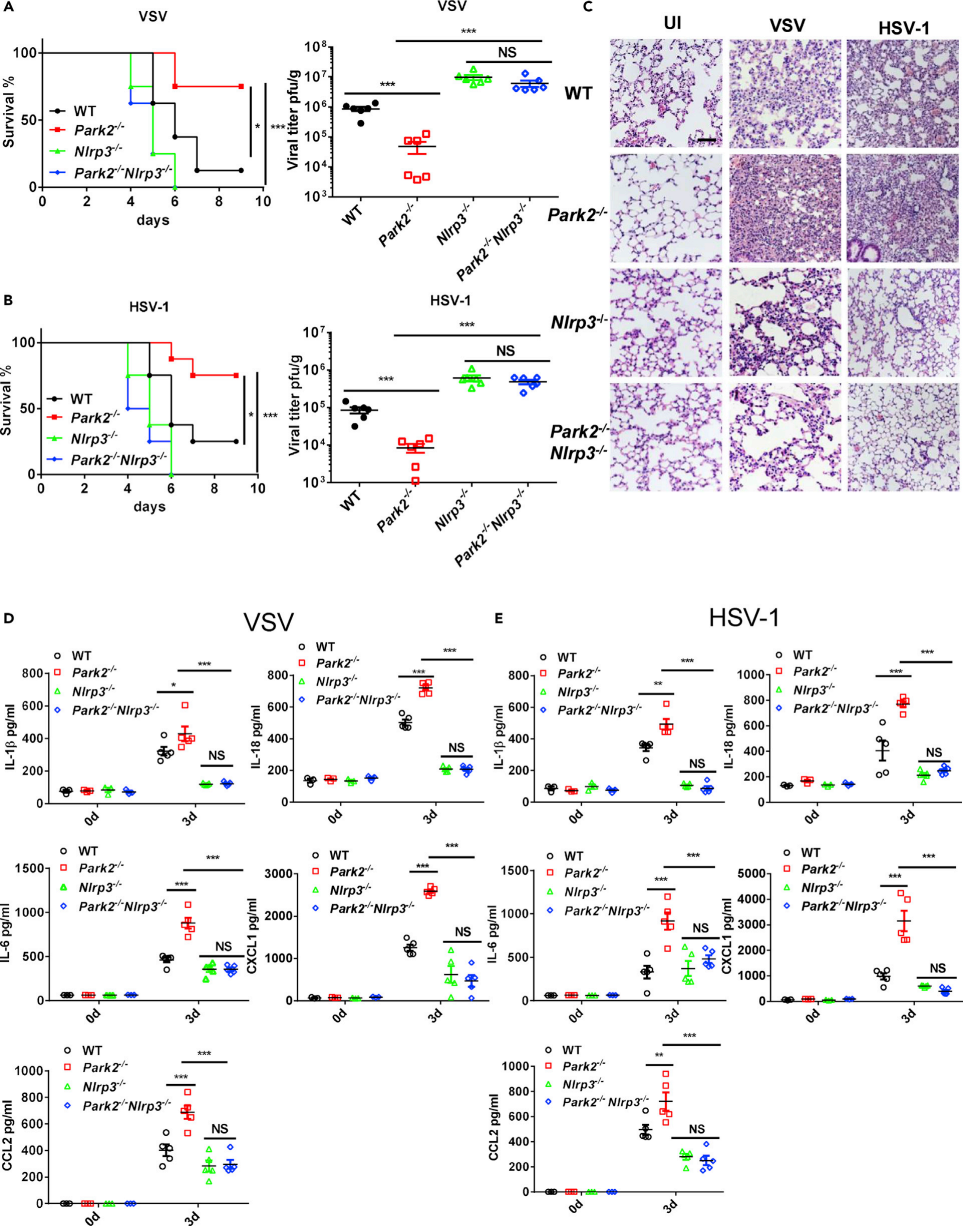
NLRP3 Deficiency Reverses the Enhanced Antiviral Inflammation and Viral Clearance in Parkin-Deficient Mice (A) WT, *Park2*^*−/−*^, *Nlrp3*^*−/−*^, and *Park2*^*−/−*^*Nlrp3*^*−/−*^ mice (n = 8 mice per group) were intranasally infected with VSV at 2 × 10^8^ plaque-forming unit (PFU) per mouse or intravenously infected with HSV-1 at 6 × 10^7^ PFU per mouse, and then the survival rates of mice are observed and recorded on the indicated days. (B) Viral titers of lungs from WT, *Park2*^*−/−*^, *Nlrp3*^*−/−*^, and *Park2*^*−/−*^*Nlrp3*^*−/−*^ mice were determined by standard plaque assay 5 days after infection as indicated in (A) (n = 6 mice per group). (C) Hematoxylin-eosin staining of lung sections from WT, *Park2*^*−/−*^, *Nlrp3*^*−/−*^, and *Park2*^*−/−*^*Nlrp3*^*−/−*^ mice on day 3 after VSV or of HSV-1 infection. (n = 3). Scale bar, 100 μm. (D and E) ELISA analysis of IL-1β, IL-18, IL-6, CXCL1, and CCL2 of BALF (D) or serum (E) from WT, *Park2*^*−/−*^, *Nlrp3*^*−/−*^ and *Park2*^*−/−*^*Nlrp3*^*−/−*^ mice on day 3 after VSV or of HSV-1 infection (n = 5 per group for viral infection, n = 3 per group for mock infection). Data are pooled from three independent experiments. Error bars show means ± SEM. *p < 0.05, **p < 0.01, ***p < 0.001, NS, not significant. Log rank (Mantel-Cox) test for survival rates, unpaired t test for viral titers and two-way ANOVA with Tukey's multiple comparisons test for ELISA.

It is clear from the above *ex vivo*-based results that Parkin deletion augments virus-induced inflammation through the activation of mtROS-NLRP3 axis. To further explore whether the enhanced survival and viral clearance in Parkin-deficient mice are dependent on NLRP3-activated antiviral inflammation, we next performed infection studies in Parkin and NLRP3 double knockout mice. Notably, we observed that like *NLRP3*^*−/−*^ mice, *Park2*^*−/−*^*Nlrp3*^*−/−*^ mice exhibited substantial reduction in survival and higher viral loads in lung after VSV (Figure 7A) and HSV-1 (Figure 7B) challenge compared with WTs, and this is markedly in contrast to the protected phenotype of *Park2*^*−/−*^ mice. Histological analysis revealed that there was much less infiltration of immune cells in lungs of *Park2*^*−/−*^*Nlrp3*^*−/−*^ mice compared with WT and *Park2*^*−/−*^ mice on day 3 after viral infection (Figure 7C), indicating the impairment of antiviral inflammation in *Park2*^*−/−*^*NLRP3*^*−/−*^ mice. In accordance with these results, VSV- (Figure 7D) and HSV-1- (Figure 7E) induced production of cytokines and chemokines, including IL-1β, IL-18, IL-6, CXCL1, and CCL2, were significantly inhibited in BALF or sera from *Park2*^*−/−*^*Nlrp3*^*−/−*^ mice, further suggesting an absolute NLRP3 dependence for the enhanced antiviral inflammation in *Park2*^*−/−*^ mice. Altogether, these results clearly demonstrate that loss of Parkin in mice promotes viral clearance through NLRP3-mediated antiviral inflammation.

### Parkin Gene Expression Is Downregulated in PBMCs from Virus-Infected Patients

To further determine the relationship of Parkin with virus infection, we analyzed by qPCR transcriptional changes in the gene expression of Parkin in BMDMs after virus infection. Unlike upregulated expression of antiviral cytokines, the expression of Parkin and another mitophagy regulator Pink1 was markedly reduced in response to VSV or HSV infection (Figure S6A), and this may serve to remove a negative regulator of antiviral inflammation and facilitate an enhanced antiviral response. This is also fully consistent with our earlier data showing that Parkin expression was downregulated at protein level after viral infection (Figure 1G). To further explore the relationship of decreased Parkin expression following viral infection with IFN-β, we treated WT MEFs and BMDMs with IFN-β. However, the Parkin expression did not decrease, suggesting the independent role of type I IFNs in regulating Parkin expression after viral infection (Figures S8A and S8B). Thus, how Parkin expression is regulated by host cells following viral infection remains to be investigated in future. To further explore the relevance of Parkin in the context of clinical infections, we next sought to assess Parkin expression in white blood cells of humans with virus infection. We isolated PBMCs from uninfected healthy donors and patients infected with hepatitis C virus (HCV, an RNA virus). qPCR analysis showed that Parkin gene expression was significantly decreased in PBMCs from virus-infected patients compared with healthy counterparts (Figure S6B). Thus, these data further support a critical regulatory role of Parkin in controlling the human response to viral infections.

## Discussion

Increasing evidence has demonstrated that mitochondria play broad roles in innate immune response, including their functioning as signal transduction platforms, providing energy and metabolites for inflammation, and generating ROS as modulators of immune response (Weinberg et al., 2015, West et al., 2011). Mitophagy is a specific form of autophagy that can remove damaged mitochondria and maintain mitochondrial homeostasis (Kubli and Gustafsson, 2012). Some studies have reported that mitophagy can control inflammasome-related inflammation by mediating the clearance of damaged, mtROS-generating mitochondria (Nakahira et al., 2011, Zhong et al., 2016). We now provide a major insight into the physiological role of mitophagy and its regulation of inflammasome-mediated inflammation in the context of antiviral immunity.

Parkin, a central player in mitophagy, can be recruited to damaged mitochondria and ubiquitylate OMM proteins to initiate mitophagy (Narendra et al., 2008). A recent study showed that Parkin recruits LUBAC to mitochondria via interaction with MAVS and inhibits RLR-MAVS signaling (Khan et al., 2016), whereas another recent study reported the converse result that Parkin fails to interact with MAVS but regulates antiviral signaling by controlling TRAF3 stability (Xin et al., 2018). However, these studies were restricted to cell-based infection models and lacked data to evaluate the physiological or pathophysiological relevance of Parkin in viral infections. We now provide insight into the role of Parkin in the *in vivo* response to viral infections. We discover a critical role for Parkin in regulating viral clearance in *vivo*. Parkin deficiency enhances mouse survival in cases of infection with RNA and DNA viruses, and this is associated with improved viral clearance. Intriguingly, Parkin deficiency did not affect early virus-induced expression of type I IFN production either *in vivo* or in primary cells and the early signaling pathways, like IRFs, that act as upstream drivers of IFN induction, were also intact in Parkin knockout cells. This contrasts with previous reports using cell models, which proposed Parkin to be a negative modulator of virus-induced type I IFN production. It is possible that this difference might be caused by using carcinoma cell lines in previous studies. A recent study demonstrated that Parkin controlled mtDNA release into cytosol and cGAS-STING-dependent activation of IFN following exhaustive exercise (Sliter et al., 2018). Although during viral infection *Park2*^*−/−*^ cells exhibit the increased release of ox-mtDNA (Figure 5D), much more nuclear acids from virus can mask the effect of more mtDNA release by Parkin deficiency on cGAS-sting activation, which might explain why Parkin does not regulate mtDNA-cGAS-STING axis-mediated IFN production after viral infection.

Although our studies question the role, if any, of Parkin in regulating the type I IFN pathway, we clearly highlight its function in controlling inflammasome-mediated antiviral immunity. Several recent studies have described a role for the NLRP3 inflammasome in mediating innate immunity to virus. In all of these cases, NLRP3 promotes antiviral inflammation and viral clearance (Allen et al., 2009, Ichinohe et al., 2009, Thomas et al., 2009). Moreover, a study in IL-1R-deficient mice has shown that the animals have reduced acute airway inflammation associated with influenza virus infection and have significantly decreased survival (Schmitz et al., 2005). A recent study suggested that inflammasome activation can negatively regulate IFN-β response to DNA viruses through caspase-1-mediated cleavage of cGAS (Wang et al., 2017), whereas our studies did not find that virus-induced IFN-β was affected in Nlrp3-deficient BMDM (Figures S7A and S7B). We reasoned that the other inflammasome complex, for example AIM2, but not NLRP3, might be involved in this regulatory process. Our present studies indicate that Parkin deficiency increases virus-induced NLRP3 inflammasome activation and related inflammation through defective mitophagy. Impaired mitophagy results in overproduction of mtROS, which is correlated with increased antiviral inflammation *in vivo*, better viral clearance, and decreased mortality in mice. Notably, recent studies clearly demonstrate that in macrophages, the Parkin-p62-mitophagy pathway can dampen NLRP3 inflammasome activation by NLRP3 agonists by the removal of damaged mitochondria to control mtROS overproduction (Zhong et al., 2016). We now discover an important role for the mitophagy-inflammasome signaling axis in the context of antiviral immunity. We propose that in response to viral infection, mtROS is produced by damaged and depolarized mitochondria leading to NLRP3 activation and antiviral inflammation that is driven by IL-1β and IL-18. However, Parkin suppresses this antiviral response by promoting the clearance of damaged mitochondria by the process of mitophagy. Notably we show that under conditions of viral infection in both experimental and clinical cases the expression of Parkin is suppressed by the virus, thus relieving a braking system that will lead to accumulation of damaged mitochondria, high levels of mtROS and NLRP3 activation, and the triggering of NLRP3-driven antiviral inflammation. It is interesting to speculate that the negative role of Parkin in antiviral inflammation may fill a physiological function ensuring that persistent infection does not trigger a dysregulated inflammatory response and pathological tissue injury.

PD is associated with accumulation of damaged and defective mitochondria, which can be the major cause of neuroinflammation (Winklhofer and Haass, 2010). Indeed, although parkinsonism exhibits age-related degeneration of dopaminergic neurons, increasing evidence show that excessive inflammation may exacerbate neuronal cell death and disease development (Wang et al., 2015). In addition, it has been suggested that viruses, and in particular influenza virus, can be a precipitating factor in the development of PD (Jang et al., 2009). We found that viral infections in humans were associated with significant decreases in the expression levels of Parkin. Given that some genetic forms of PD are associated with mutations in the Parkin gene, virus-induced suppression of Parkin may phenocopy the pathology associated with Parkin-related PD. Indeed, impaired mitophagy resulting from Parkin deficiency or virus-induced suppression may link excessive NLRP3 inflammasome activation in microglia to chronic neuroinflammation that exacerbates neuronal cell death. Thus the present studies strongly prompt future studies to explore the potential role of Parkin in virus-related PD.

Taken together, our findings demonstrate a vital role for Parkin and mitophagy in regulating antiviral inflammation. These pathways act as a brake on virus-induced NLRP3 inflammasome activation by controlling mtROS. Our studies thus promote Parkin as a potential therapeutic target in the promotion of antiviral immunity.

### Limitations of the Study

Our study clearly demonstrates that Parkin plays an important role in antiviral immunity by controlling mtROS-NLRP3 axis-mediated inflammation. The decreased expression of Parkin following viral infection is observed in mice and humans, but how Parkin expression is regulated by host cells following viral infection remains unknown. The known PD-related Parkin mutants majorly include splice site mutations and missense mutations. Our study in Parkin-deficient mice can well reflect the effect of slice site mutants on antiviral response. However, we do not know the relationship of antiviral response with missense mutations, which will lead to a loss of Parkin function in E3-ligase catalytic activity. Thus, these issues need to be investigated in future studies.

## Methods

All methods can be found in the accompanying Transparent Methods supplemental file.

## Supplemental Information

Document S1. Transparent Methods and Figures S1–S8
