# Supplementary material for: Parkin Impairs Antiviral Immunity by Suppressing the Mitochondrial Reactive Oxygen Species-Nlrp3 Axis and Antiviral Inflammation
Source: iScience. 2019 Jun 11;16:468–84. doi: 10.1016/j.isci.2019.06.008 (PMC6593176; doi:10.1016/j.isci.2019.06.008)
Supplement: Document S1. Transparent Methods and Figures S1–S8 [file mmc1.pdf]

ISCI, Volume *16*

Supplemental Information

Parkin Impairs Antiviral Immunity by Suppressing the Mitochondrial Reactive Oxygen Species-Nlrp3 Axis and Antiviral Inflammation

Jian Li, Chunmei Ma, Fei Long, Dongxue Yang, Xue Liu, Yingchao Hu, Chunyan Wu, Bingwei Wang, Min Wang, Yunzi Chen, Genyan Liu, Paul N. Moynagh, Jiawei Zhou, Tao Peng, and Shuo Yang

Supplementary information

Transparent methods

Mice

Park2$^{-/-}$ mice (Strain ID: 006582, C57BL/6 background) were provided by Dr. Jiawei Zhou from the Jackson Laboratory. *Nlrp3*$^{-/-}$ mice on C57BL/6 background were a gift from Dr. V. Dixit (Genentech). *Park2*$^{-/-}$ *Nlrp3*$^{-/-}$mice were established by crossing *Nlrp3*$^{-/-}$ with *Park2*$^{-/-}$mice. Mice used in this study (8-10-week-old male mice) were kept and bred under specific-pathogen-free conditions. All experiments were performed under protocols approved by the Institutional Animal Care and Use Committee of Nanjing Medical University, Guangzhou Medical University and Shanghai Institute of Neuroscience, Chinese Academy of Sciences.

Reagents

IL-18 ELISA capture antibody (D047-3), detection antibody (D048-6) and standard protein (12405-1) were from R&D; IFN-β ELISA capture antibody (sc-57201) was from Santa Cruz; IFN-β detection antibody(32400-1) and standard protein (12400-1) were from R&D. Anti-P62(5114), anti-LC3B(2775), anti-myc(2276), anti-phosphorylated IRF3(4947s), anti-IRF3(4302s), anti-TBK1(3013s), anti-phosphorylated TBK1(5483s), anti-phosphorylated IκBα(9246s) and anti-phosphorylated P38(9212s) were from Cell Signaling Technology; Anti-flag (F3165), anti-β-actin(AC-15; A 1978) and anti-α-tubulin(T9026) were from Sigma; Anti-Parkin (sc-32282) and anti-Traf3(sc-949) were from Santa Cruz; Anti-IL-1 β (AB-401-NA) was from R&D; Anti-caspase1 (AG-20B-0042-C100) was from Adipogen. Anti-mouse-HRP and anti-rabbit-HRP were from Jackson ImmunoResearch. Anti-CD45-AF700(30-F11,85-11-0112-81), anti-CD11b-FITC(M1/70,85-12-0114-81), anti-CD11c-PE (N418, 48-9688-82), anti-Ly6C-PE-Cy7(HK1.4,25-5932-82), anti-Ly6G- eFlour450 (1A8-LY6G, 48-9668-82), anti-TCRβ−eFlour450(H57-597, 48-5961-80), anti-B220-Percp-Cy5.5 (Ra3-6B2,45-0452-82) and anti-NK1.1-PE-Cy7(PK136, 25-5941-81) antibodies were from eBioscience; MHC II (M5/114.15.2, 107627.0) was from Biolegend. Pam3CSK4 (tlrl-pms) and Poly(I:C) (tlrl-picw) were from Invivogen. Mito-Q was from MCE(HY-100116). Ficoll was from GE Health (45-001-749).

Viruses

VSV (Indiana strain) and HSV-1 (KOS strain) were obtained from Dr. Yichuan Xiao (Shanghai Institutes for Biological Sciences, China). VSV-GFP and HSV-1-GFP were from Dr. Tao Peng. All viruses were amplified by infection of a monolayer of African green monkey kidney cells (Vero). Briefly, 24-48 h after infection, the infected cells were frozen and thawed for three times to release virus. Then the cell lysate and culture supernatant were harvested and clarified by centrifugation. Viral titers were determined by standard plaque assay on confluent monolayers of Vero cells.

Viral infection in vivo

Age matched male mice (8-10-week old) were intranasally infected with VSV at 2×10^8 pfu per mouse or intravenously infected with HSV-1 at 6×10^7 pfu per mouse. Lungs from inoculated animals were homogenized in PBS and assayed for viral loads by plaque assay on Vero cells. Serum, BALF and lungs were also collected for Elisa, histology and FACS analysis.

Plaque Assay for Virus Titers

Viral infectivity was quantified using Vero cell monolayer. In brief, several 10-fold serial dilutions of the samples were made, added to Vero cell monolayer and incubated for 1–2 days. Then the cells were fixed and stained with 0.1% crystal violet solution for 10 min. Plaques were counted and viral titers were determined as plaque-forming units per ml by multiplying the dilution factor. For assay of viral load in mouse organs, after mice were sacrificed organs were collected individually, disrupted with a tissue homogenizer, frozen, and thawed three times to release the virus. Serial dilutions were made of the organ homogenates and then subjected to plaque assay.

Cell preparation and stimulation

MEFs were generated from E12-14 embryos, maintained in DMEM supplemented with 10% FBS, and sub-cultured no more than five passages before experiments. For isolation of BMDMs and BMDCs, tibias and femurs were removed from mice by sterile techniques and bone marrow was flushed with fresh medium. BMDMs were cultured in DMEM supplemented with 10% FBS in the presence of 10% L929 conditioned medium. BMDCs were cultured in RPMI-1640 complemented with 10% FBS in the presence of 10% J558 culture medium. After 5 days culture, 1.5×10^6 BMDMs and BMDCs were plated overnight in 12-well plates and were infected with VSV and HSV-1 as indicated in legends. For inducing inflammasome activation, BMDMs were primed with 1μg/mL Pam3CSK4 for 3 h and then stimulated with ATP (2.5 mM) for 1 hour or with VSV and HSV-1 as indicated MOI for 24 h. THP-1 cells were cultured in RPMI-1640 medium plus GlutaMAX-I medium (Gibco) and transfected with control or Parkin-specific siRNA for 48hr. The following siRNA sequences were used: Con-siRNAs UUCUCCGAACGUGUCACGUTT, Con-siRNAas ACGUGACACGUUCGGAGAATT; Park2-siRNAs CGACCCUCAACUUGGCUACTT, Park2-siRNAas GUAGCCAAGUUGAGGGUCGTT. The knockdown cells were then infected with VSV and HSV-1 for indicated times.

Immunoblotting

Cells were grown in 12-well plates and after treatment were then collected in 120μl NP-40 lysis buffer (50 mM Tris-HCl, pH 7.4, containing 150 mM NaCl, 1% (vol/vol) Igepal, 10% (wt/vol) glycerol, 50 mM NaF, 1 mM Na3VO4, 1 mM dithiothreitol, 1 mM phenylmethylsulphonyl fluoride and complete protease-inhibitor 'cocktail' (Sigma)), followed by incubation for 30 min at 4 °C. Cell lysates were initially precleared by centrifugation at 14000 rpm. An aliquot of 6X SDS-PAGE sample buffer (1M Tris-HCl, pH 6.8, 20% (w/v) glycerol, 4% (w/v) SDS, 3% (w/v) DTT and 0.02% (w/v) bromophenol blue) was added to cell lysates. Samples were resolved by SDS-PAGE, transferred to nitrocellulose membranes and analyzed by immunoblot with the appropriate antibodies. Immunoreactivity was visualized by the Odyssey Imaging System (LI-COR Biosciences) or enhanced chemiluminescence.

Measurement of cytosolic mtDNA

WT and *Park2*$^{-/-}$ MEFs were infected with VSV and HSV-1 (MOI=10) for 4h.Total DNA was isolated from the indicated cells using E.Z.N.A.™ MicroElute Genomic DNA kit (OMEGA) according to manufacturer's instructions. mtDNA was quantified by qPCR using primers specific for the mitochondrial D-loop region or a specific region of mtDNA that is not inserted into nuclear DNA (non-NUMT). Nuclear DNA encoding Tert and B2m was used for normalization.

D-loop F: 5′AATCTACCATCCTCCGTGAAACC3′;
D-loop R: 5′TCAGTTTAGCTACCCCCAAGTTTAA3′
Tert F: 5′CTAGCTCATGTGTCAAGACCCTCTT3′
Tert R: 5′GCCAGCACGTTTCTCTCGTT3′
B2m F: 5′ATGGGAAGCCGAACATACTG3′
B2m R: 5′CAGTCTCAGTGGGGGTGAAT3′
non-NUMT F: 5′CTAGAAACCCCGAAACCAAA3′
non-NUMT R: 5′CCAGCTATCACCAAGCTCGT3′

Measurement of cytosolic 8OH-dG

WT and *Park2*$^{-/-}$ BMDMs were primed with 1μg/mL Pam3CSK4 for 3 hr and then stimulated with VSV and HSV-1(MOI=10) for 5h. Cellular fractionation was then performed using a mitochondrial isolation kit for cultured cells according to manufacturer's instructions. Cytosolic protein concentration and volume of the supernatant were normalized, DNA was isolated from 300ul cytosolic fractions using E.Z.N.A.™ MicroElute Genomic DNA kit according to manufacturer's instructions. The 8OH-dG was quantified using 8OH-dG ELISA kit (Elabscience), as the manufacturer's instruction.

RT-qPCR assays

Total RNA was extracted with Trizol reagent (Invitrogen) and reversed-transcribed with Reverse Transcription System (Vazyme). Reverse transcription products of different samples were amplified by Steponeplus (Applied Biosystems) using Brilliant SYBR Green QPCR Master mix (Vazyme) according to the manufacturer's instructions and data were normalized by the level of HPRT expression in each individual sample. $2^{-\Delta\Delta Ct}$ method was used to calculate relative expression changes. Heatmap analysis was carried out using MeV Experiment View software. The following primers were used:

Mouse *Il1b*	S	As
	5 ATGCCACCTTTTGACAGTGATG 3	5 GTTGATGTGCTGCTGCGAGA 3
Mouse *Il6*	S	As
	5 CTTGGGACTGATGCTGGTGAC 3	5 GCCATTGCACAACTCTTTTCTC 3
Mouse *Il18*	S	As
	5 TCAAAGTGCCAGTGAACCCC 3	5 AGGGTCACAGCCAGTCCTCTT 3
Mouse *Il12*	S	As
	5 TGGTTTGCCATCGTTTTGC 3	5 GGGAGTCCAGTCCACCTCTACA 3
Mouse *Irf7*	S	As
	5 CAGCACAGGGCGTTTTATCTT 3	5 TCTTCCCTATTTTCCGTGGC 3
Mouse *Isg15*	S	As
	5 AGCAGATTGCCCAGAAGATTG 3	5 CCCCTTTCGTTCCTCACCA 3
Mouse *Ifna*	S	As
	5 CTTTCCTCATGATCCTGGTAATGAT 3	5 AATCCAAAATCCTTCCTGTCCTTC 3
Mouse *Ifnb*	S	AS
	5 CCCTATGGAGATGACGGAGA 3	5 CCCAGTGCTGGAGAAATTGT 3

Mouse *Ndufa6*	S	AS
	5ACGGCAGCGAGTCCCGTGGCACCGC3	5 GTGTCCGCTGCTTCCATACTT 3
Mouse *Ndufab1*	S	AS
	5TGGGTTGGCGTAGCCATGGCGTCTC 3	5 TCGGTCCTTGATTCCGTCTAAC 3
Mouse *Hprt*	S	AS
	5 GTCCCAGCGTCGTGATTAGC 3	5 TGGCCTCCCATCTCCTTCA 3
Mouse *Ddx58*	S	AS
	5 CACATTTGCGGAAATACAACG 3	5 TGCTGCTTCTCGGACATCG 3
Moues *Isg15*	S	AS
	5 AGCAGATTGCCCAGAAGATTG 3	5 CCCCTTTCGTTCCTCACCA 3
Mouse *Stat1*	S	AS
	5 TCACAGTGGTTCGAGCTTCAG 3	5 GCAAACGAGACATCATAGGCA 3
Human Park2	S	AS
	5 ATGATAGTGTTTGTCAGGTTCAACT 3	5 CCCTGTCGCTTAGCAACCA 3
Human Pink1	S	AS
	5ATGGCGGTGCGACAGGCGCTGGGCC3	5 CGGCTTGCTTTTCTGGGTAA 3
Human Hprt	S	AS
	5 GGCGATGTCAATAGGACTCCAGAT 3	5 GGTGAAAAGGACCCCACGAA3

ELISA

Primary mouse cells were stimulated as indicated. Conditioned media were collected and measured for levels of IL-1β (DY401), TNF-α (DY410) and IL-12 (DY2398) according to manufacturer's instructions (R&D Systems). The level of IL-18 and IFN-β was assayed by an in-house sandwich ELISA system. The serum, BALF and lung from wild-type and *Park2*$^{-/-}$ mice were collected after infection as indicated and the levels of IL-1β, IL-18, IL-6(DY406), CXCL1 (DY453) and CCL2 (DY479) in these samples were measured by sandwich ELISA (R&D Systems). Conditioned supernatants from human THP-1 siRNA-transfected cells were assayed for IL-1β (DY201, R&D), IL-6 (DY206, R&D), IL-18(70-EK1181, Multi Sciences) and IFN-β (70-EK1236-48, Multi Sciences).

Cytotoxicity Assay (LDH)

Conditioned medium from treated BMDMs was assessed for LDH release using Pierce LDH Cytotoxicity Assay Kit (Promega) according to the manufacturer's instructions. Relative LDH release was calculated as LDH release [%] = 100 * (measurement – unstimulated control)/ (lysis control – unstimulated control).

Fluorescence and Confocal microscopy

BMDMs were seeded on slides overnight and then were stimulated as indicated. After being stained with TMRM (20 nM) in the dark for 30 minutes at 37 °C, the cells were fixed, washed three times with PBS and then followed by Hoechst for 5 min. Images were captured using Nikon50i fluorescent microscope.

Flow cytometry

For isolation of lung infiltrating immune cells, mice on day 3 after viral infection were anesthetized and perfused with 10-15 mL of PBS (ice cold) through the right ventricle until lungs cleared of blood. Lungs were harvested, cut into small pieces and incubated in collagenase type IV (0.5mg/ml) and DNase I (10U/ml) at 37°C under agitation (200 rpm) conditions for 90 min. The digested tissues were filtered through a 100 μm filter and the plunger end of syringe was used to push the cells over the filter. Homogeneous cell suspensions were centrifuged over a 40%/80% discontinuous Percoll gradient (GE Healthcare), mononuclear cells were isolated from the interface. The cells were suspended in PBS containing 2% (w/v) FBS. Once lysed in RBC lysis buffer, cells were washed with the suspension buffer and stained with fluorochrome-conjugated surface marker antibodies for FACS analysis. The following antibodies were used: CD45, CD11b, ly6C, ly6G, MHC II^{+}, CD11c, TCRβ, B220 and NK1.1. Mitochondria-associated ROS levels were measured by staining cells with MitoSOX (Invitrogen) at 2.5 μM for 30 min at 37 °C. Cells were then washed with PBS solution and re-suspended in cold PBS solution containing 1% FBS for FACS analysis. All flow cytometry was performed on an Attune NxT flow cytometer (Thermofisher) and data were analyzed by FlowJo 7.6.1 software.

Histology and Immunohistochemistry

Lungs from control or virus-infected mice were fixed in 10% phosphate-buffered formalin, embedded into paraffin and sectioned. Sections of lung 5 μm in thickness were collected on superfrost slides, deparaffinized in xylol and rehydrated, and stained with hematoxylin-eosin (H&E) solution y. For immunohistochemical staining, sections were blocked and incubated with primary antibodies and horseradish peroxidase-conjugated secondary antibodies after heat induced antigen retrieval. Diaminobenzidine was used for detection. Images were captured with Nikon 50i microscope.

Analysis of PBMC samples from patients with viral infection

Individuals diagnosed with HCV infection and healthy volunteers were enrolled in this study. Clinical parameters and characterization of these patients were determined by a physician (GY. Liu). Blood samples were collected during severe illness. PBMCs were isolated by Ficoll-density gradient centrifugation (GE Healthcare). Total RNA was extracted with Trizol reagent (Invitrogen) and reversed-transcribed with Reverse Transcription System (Vazyme). This study was approved by the institution review board at the First Affiliated Hospital of Nanjing Medical University. All patient managements and blood sample collection were carried out in accordance with the relevant guidelines. The following table was information of human subjects.

Name	State	The sex	Age
Hu	HCV	female	32
Jiang	HCV	female	41
Lu	HCV	female	27
Han	HCV	female	28
Su	HCV	female	37
Sun	HCV	female	26

Wang	HCV	female	49
Lv	HCV	female	33
Liu	Healthy	female	24
Wu	Healthy	female	26
Hu	Healthy	male	25
Zhang	Healthy	male	26
Rao	Healthy	male	27
Long	Healthy	male	26
Yang	Healthy	male	23
Wang	Healthy	male	26

Statistical Analysis

Data are presented as the mean ± standard error of the mean (SEM). Samples were analyzed using unpaired t-test for two groups and ANOVA for multiple groups. Survival curves were generated via the product-limit method of GraphPad. In all cases, a P value of less than 0.05 was considered statistically significant.

Figure S1

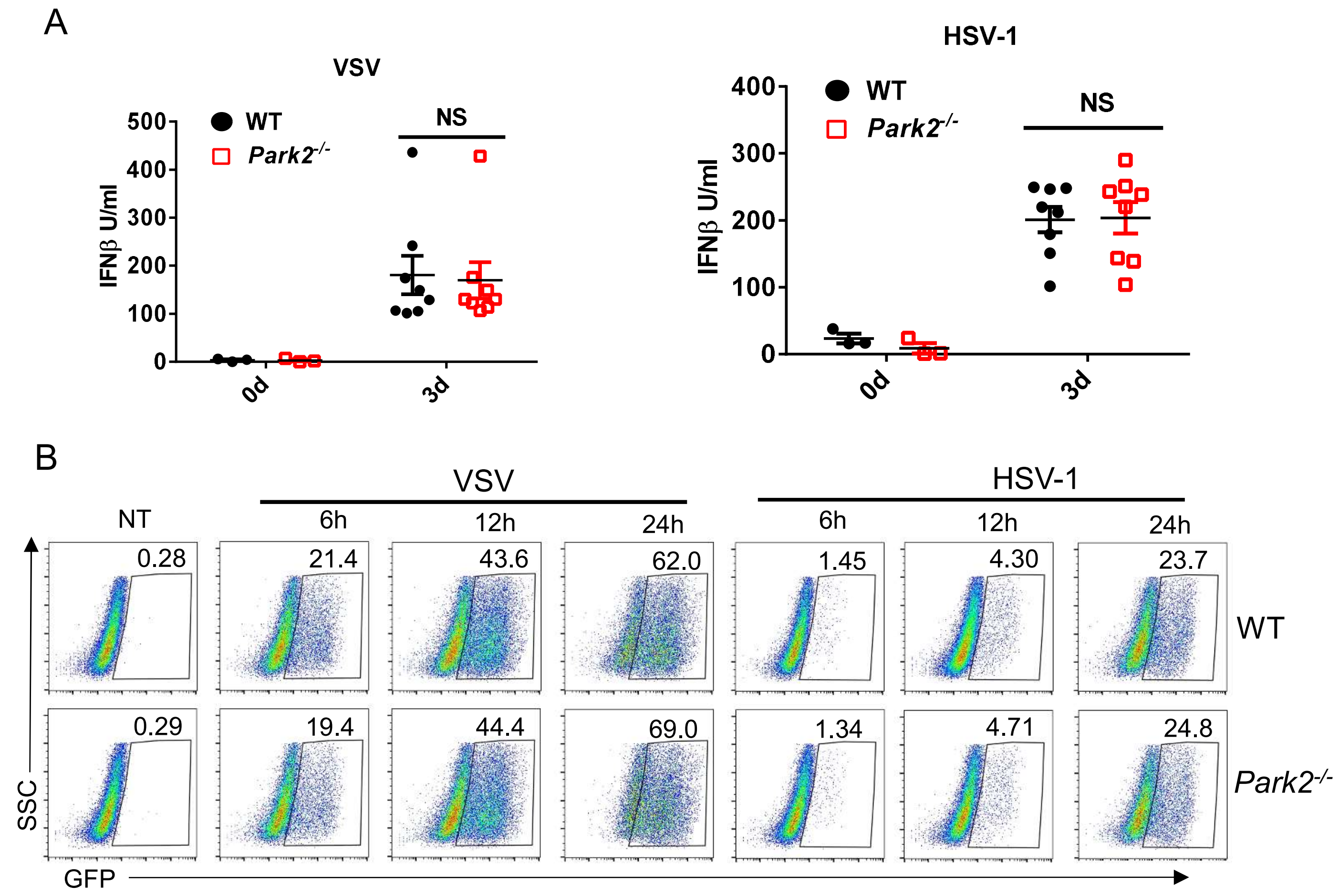

Figure S1. Loss of Parkin doesn't affect IFN production in viral-infected mice, susceptibility of MEFs to VSV or HSV-1 infection, Related to Figure 1.

(A) ELISA analysis of IFN-β of lung homogenates from WT and *Park2*$^{-/-}$ mice infected with the indicated viruses for 3 days (n=8 for viral infection, n=3 for mock infection).

(B) Flow cytometry analysis of GFP expression in WT and *Park2*$^{-/-}$ MEFs infected with VSV-GFP and HSV-1-GFP at MOI 1 for 6,12 and 24 h. NT, No treatment

Figure S2

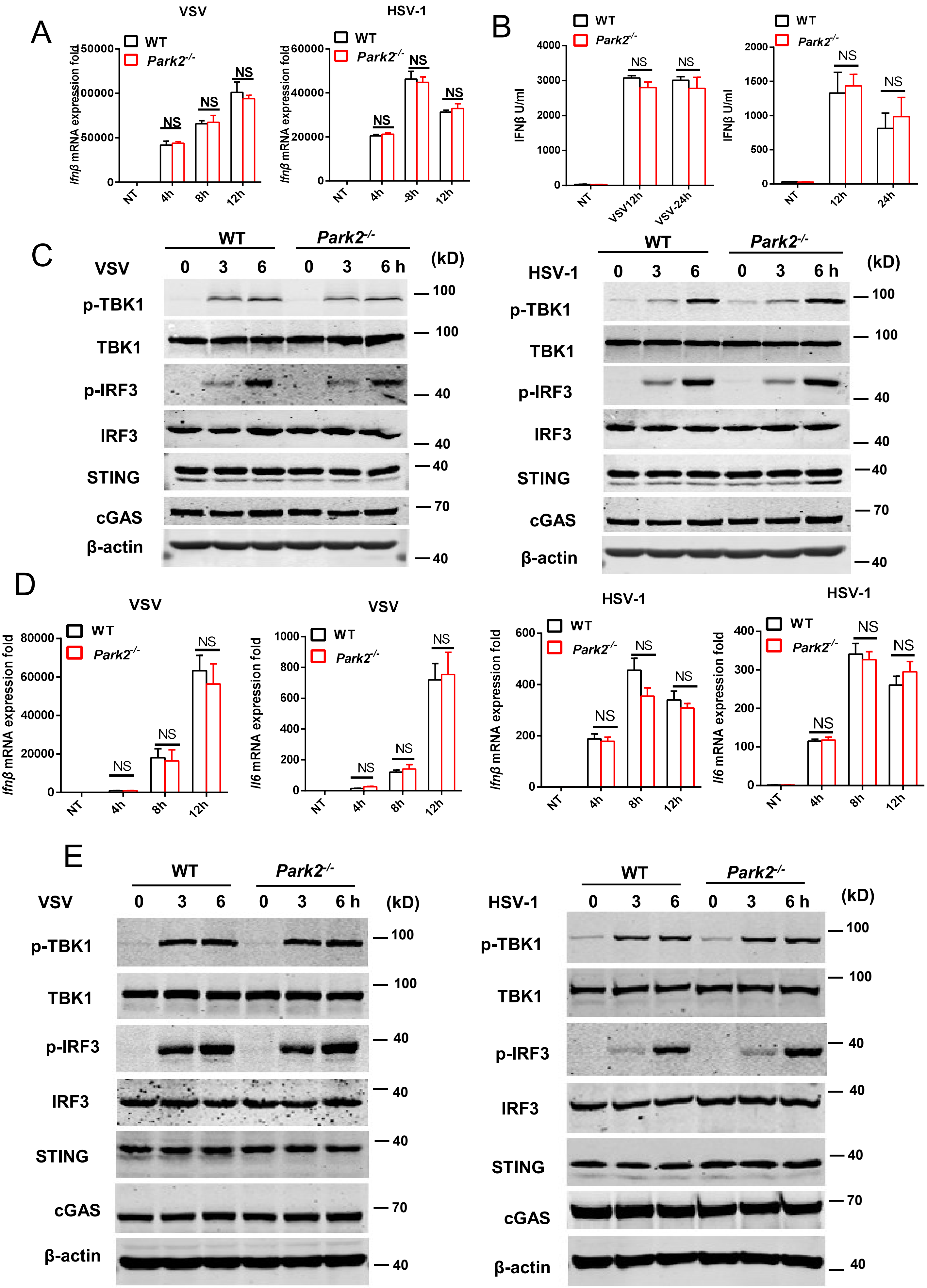

A
VSV
HSV-1
WT
Park2-/-
Ifnβ mRNA expression fold
NS
NT
4h
8h
12h
B
VSV
HSV-1
IFNβ U/ml
VSV-12h
VSV-24h
24h
C
VSV
HSV-1
0
3
6
6 h
(kD)
p-TBK1
TBK1
p-IRF3
IRF3
STING
cGAS
β-actin
100
70
40
D
Il6 mRNA expression fold
E

Figure S2. Parkin deficiency doesn't affect antiviral signaling and IFN production in BMDCs and BMDMs, Related to Figure 1.

(A) RT-qPCR analysis of *Ifnb* mRNA of WT and *Park2*$^{-/-}$ BMDCs infected with VSV or HSV-1 for 4, 8 and12h. NT, No treatment

(B) ELISA of IFN-β in the supernatants of WT and *Park2*$^{-/-}$ BMDCs infected with VSV or HSV-1 for 12 and 24h. NT, No treatment

(C) Immunoblot analysis of phosphorylated (p-) and total IRF3, STING and cGAS in WT and *Park2*$^{-/-}$BMDCs infected for 0, 3 or 6h with VSV or HSV-1.

(D) RT-qPCR analysis of *Ifnb* and *Il6* mRNA of WT and *Park2*$^{-/-}$ BMDMs infected with VSV or HSV-1 for 4, 8 and12h. NT, No treatment

(E) Immunoblot analysis of phosphorylated (p-) and total IRF3, STING, cGAS and β-actin (loading control) in WT and Park2$^{-/-}$BMDMs infected for 0, 3 or 6h with VSV or HSV-1.

Data are pooled from three independent experiments (A, B and D) or are representative of two independent experiments (C and E). Error bars show means ± SEM. NS, not significant. two-way ANOVA with Sidak's multiple comparisons test.

Figure S3

A

VSV

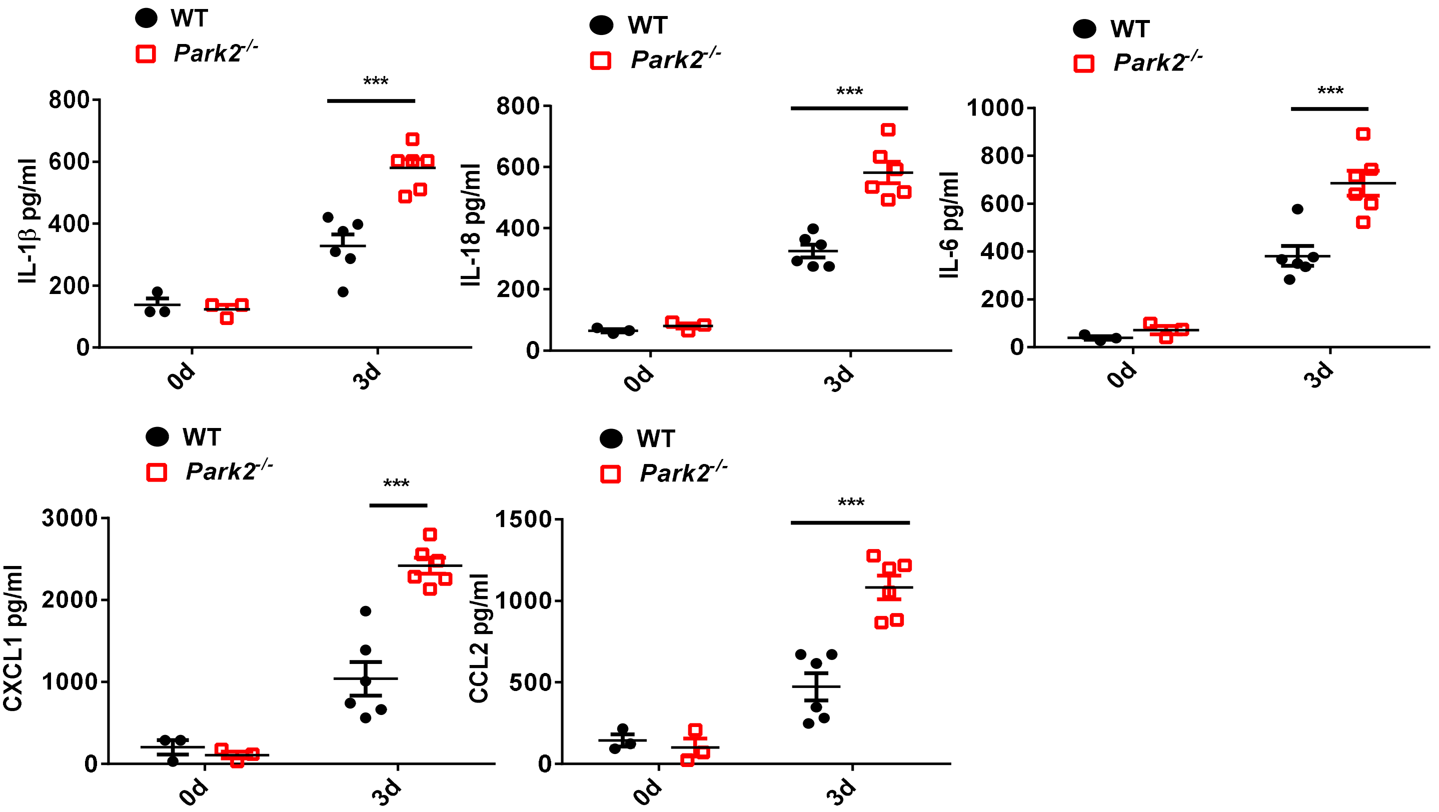

B

HSV-1

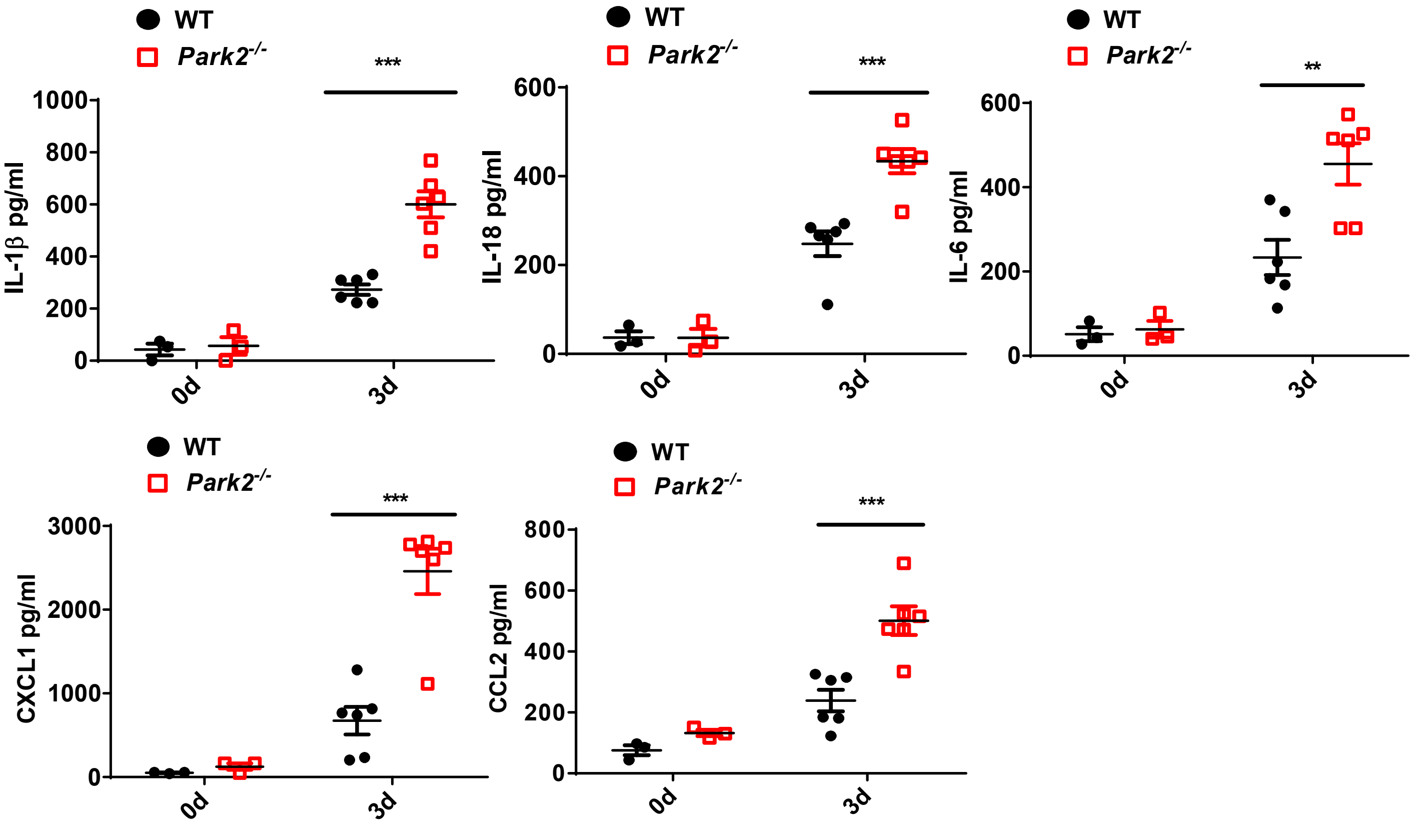

Figure S3. Parkin deficiency enhances viral-induced expression of pro-inflammatory cytokines in lung, Related to Figure 2 and 3.

(A) ELISA analysis of IL-1β, IL-18, IL-6, CXCL1 andCCL2 of lungs from WT and *Park2*$^{-/-}$mice infected with VSV for 3 days (n=6 for viral infection, n=3 for mock infection).

(B) ELISA analysis of IL-1β, IL-18, IL-6, CXCL1, CCL2 of lungs from WT and *Park2*$^{-/-}$ mice infected with HSV-1 for 3 days (n=6 for viral infection, n=3 for mock infection). Data are pooled from three independent experiments. Error bars show means ± SEM. * * $P < 0.01$, * * * $P < 0.001$. Two-way ANOVA with Sidak's multiple comparisons test.

Figure S4

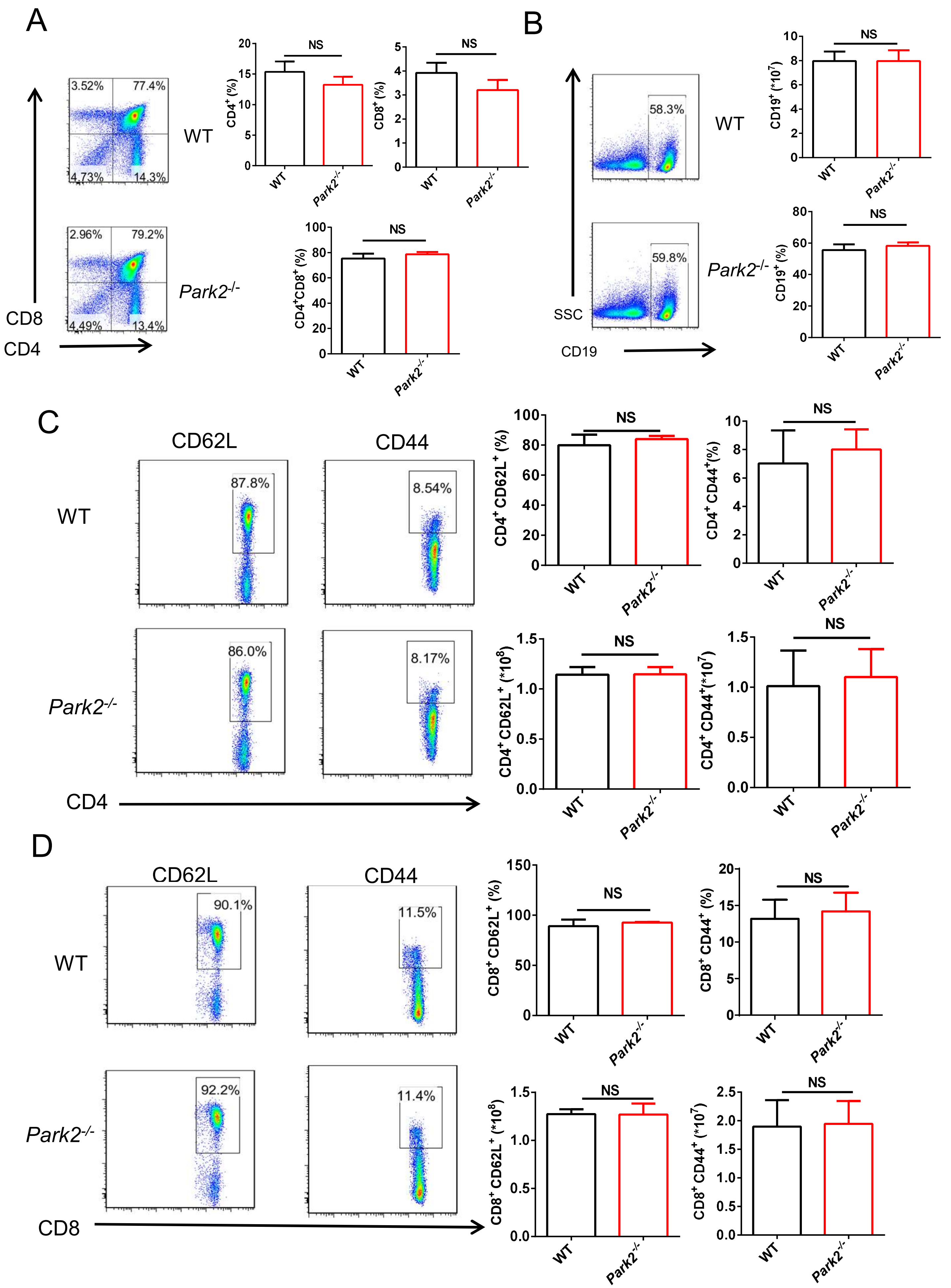
A
3.52%
77.4%
4.73%
14.3%
WT
2.96%
79.2%
4.49%
13.4%
Park2-/-
CD8
CD4
CD4+ (%)
CD8+ (%)
CD4+CD8+ (%)
NS
WT
Park2-/-
B
58.3%
WT
59.8%
Park2-/-
SSC
CD19
CD19+ (*10^7)
CD19+ (%)
NS
C
CD62L
CD44
87.8%
8.54%
86.0%
8.17%
WT
Park2-/-
CD4
CD4+ CD62L+ (%)
CD4+ CD44+(%)
CD4+ CD62L+ (*10^8)
CD4+ CD44+(*10^7)
NS
D
CD62L
CD44
90.1%
11.5%
92.2%
11.4%
WT
Park2-/-
CD8
CD8+ CD62L+ (%)
CD8+ CD44+ (%)
CD8+ CD62L+ (*10^8)
CD8+ CD44+ (*10^7)
NS

Figure S4. Parkin deficiency doesn't affect lymphoid cells, Related to Figure 2 and 3.
Flow cytometry analysis of percentages and numbers of lymphoid cells in thymus and spleen of WT or *Park2*$^{-/-}$ mice at 10 weeks of age.
(A) CD4^{+}, CD8^{+} and CD4^{+}CD8^{+} T cells in thymus.
(B) CD19^{+} B cells in spleen.
(C) CD4^{+} CD44^{+} and CD4^{+} CD62l^{+} T cells in spleen.
(D) CD8^{+} CD44^{+} and CD8^{+} CD62l^{+} T in spleen.
N=3. Error bars show means ± SEM. NS, not significant. Unpaired t test.

Figure S5

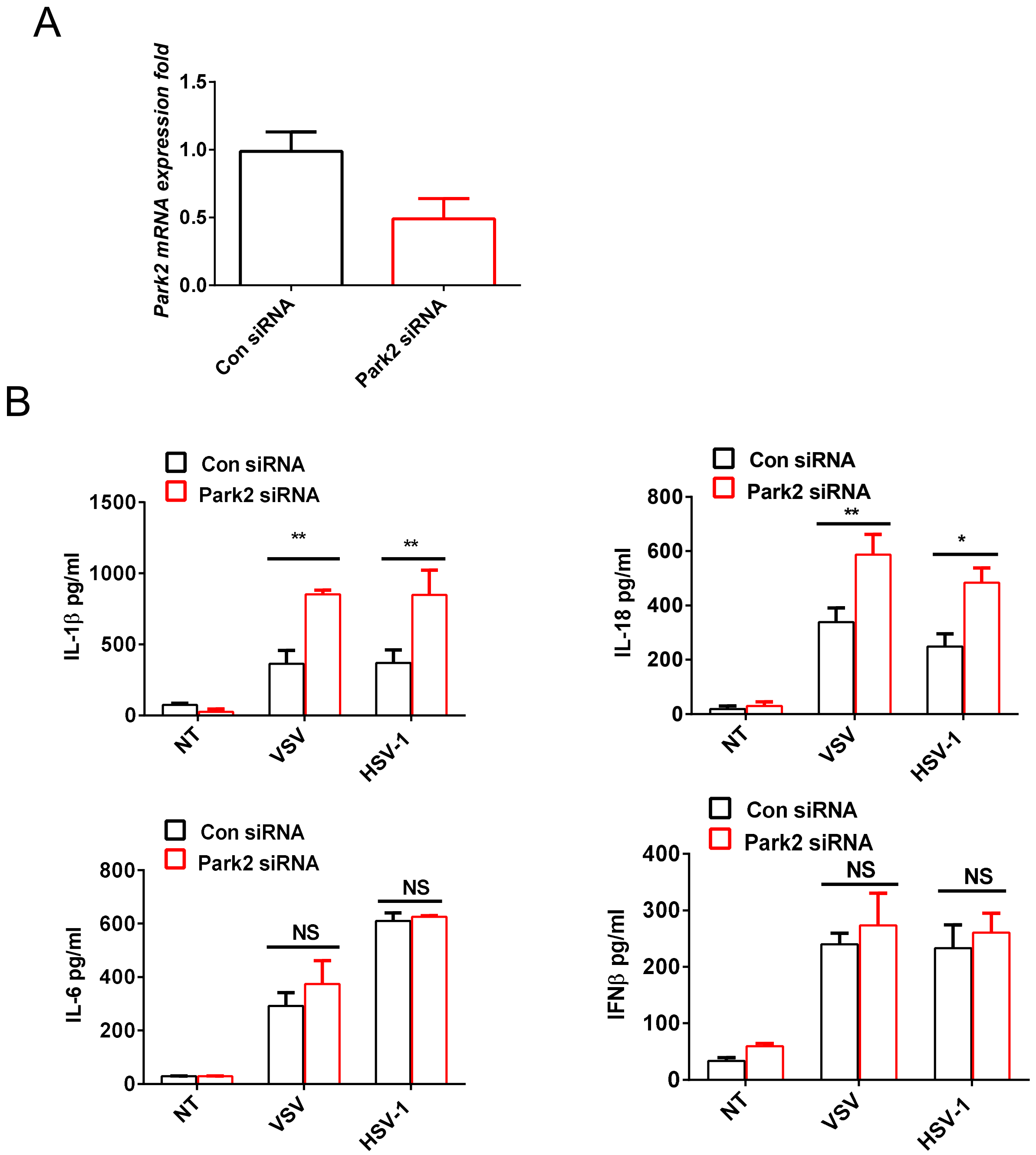

Figure S5. Parkin knockdown in THP1 cells doesn't affect IFN production but increases the production of inflammasome-related cytokines in response to viral infection, Related to Figure 4.

(A) RT-qPCR analysis of *Park2* mRNA of THP1 cells transfected with control scramble or Park2-specific siRNA for 48h.

(B) ELISA analysis of IL-1β, IL-18, IL-6 and IFN-β in the supernatants of THP1 cells transfected with control scramble or Parkin-specific siRNA for 48h and then infected with VSV at MOI 1 or HSV-1 at MOI 2 for 24h.

Data are pooled from three independent experiments. Error bars show means ± SEM. * $P < 0.05$, * * $P < 0.01$, NS, not significant. Two-way ANOVA with Sidak's multiple comparisons test.

Figure S6

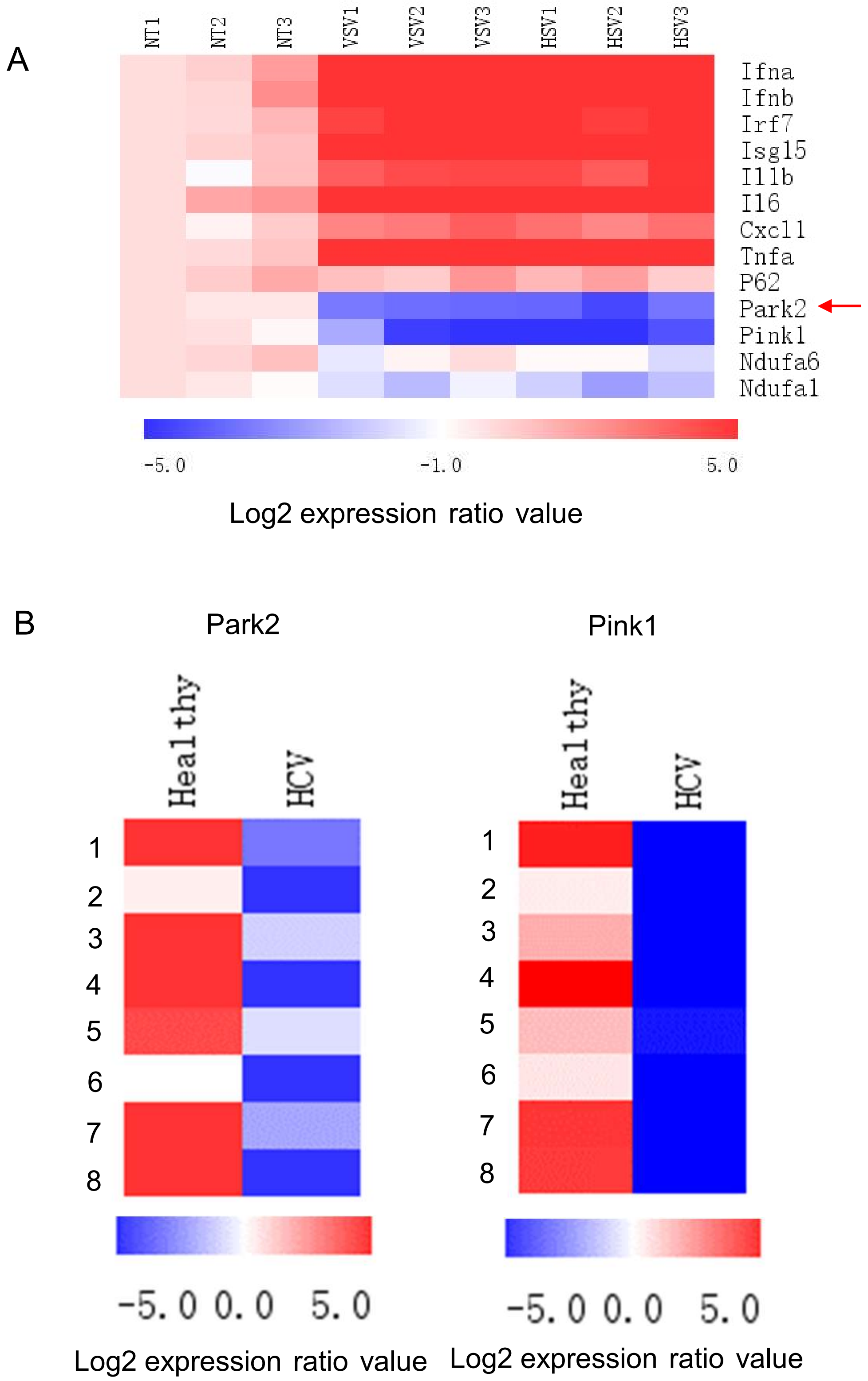
A
NT1
NT2
NT3
VSV1
VSV2
VSV3
HSV1
HSV2
HSV3
Ifna
Ifnb
Irf7
Isg15
Il1b
Il6
Cxcl1
Tnfa
P62
Park2
Pink1
Ndufa6
Ndufa1
-5.0
-1.0
5.0
Log2 expression ratio value
B
Park2
Healthy
HCV
1
2
3
4
5
6
7
8
-5.0 0.0 5.0
Log2 expression ratio value
Pink1
Healthy
HCV
1
2
3
4
5
6
7
8
-5.0 0.0 5.0
Log2 expression ratio value

Figure S6. Parkin gene decrease in response to virus infection in BMDMs and PBMCs of patients, Related to Figure 1.

(A) Heatmap visualization of gene expression for *Park2, Pink1, P62, Irf7, Isg15, Ifnb, Ifna, Il1b, Il6, Cxcl1, Tnfa, Ndufab1* and *Ndufa6* in BMDMs infected with VSV or HSV-1 for 6h determined by qPCR (log2 fold values). The abundance of each mRNA was normalized relative to PCR of the housekeeping gene hprt.

(B) RT-qPCR analysis of *Park2* and *Pink1* mRNA expression of PBMCs from healthy and HCV patients (log2 fold values).

Figure S7

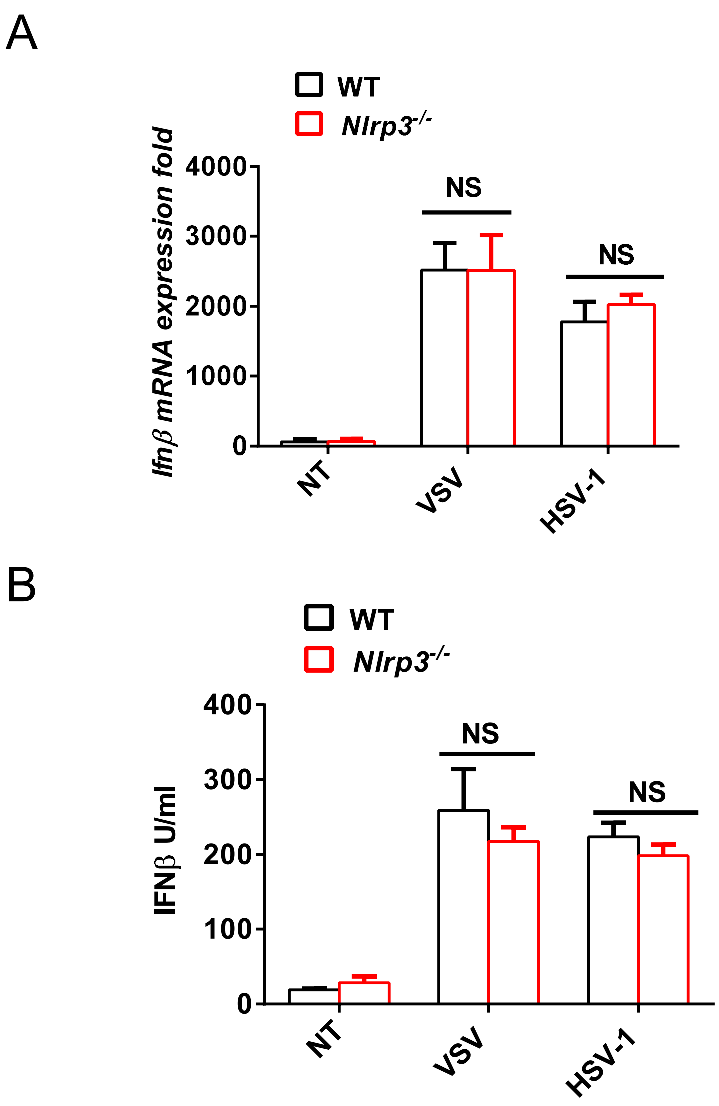

Figure S7. NLRP3 deficiency in BMDMs doesn't affect IFN production in response to viral infection, Related to Figure 6.

(A) RT-qPCR analysis of *Ifnb* mRNA of WT and *Nlrp3*$^{-/-}$ BMDCs infected with VSV or HSV-1 at MOI 1for 12h.

(B) ELISA of IFN-β in the supernatants of WT and *Nlrp3*$^{-/-}$ BMDCs infected with VSV or HSV-1 at MOI 1 for 24h.

Data are pooled from three independent experiments. Error bars show means ± SEM. NS, not significant. two-way ANOVA with Sidak's multiple comparisons test.

Figure S8

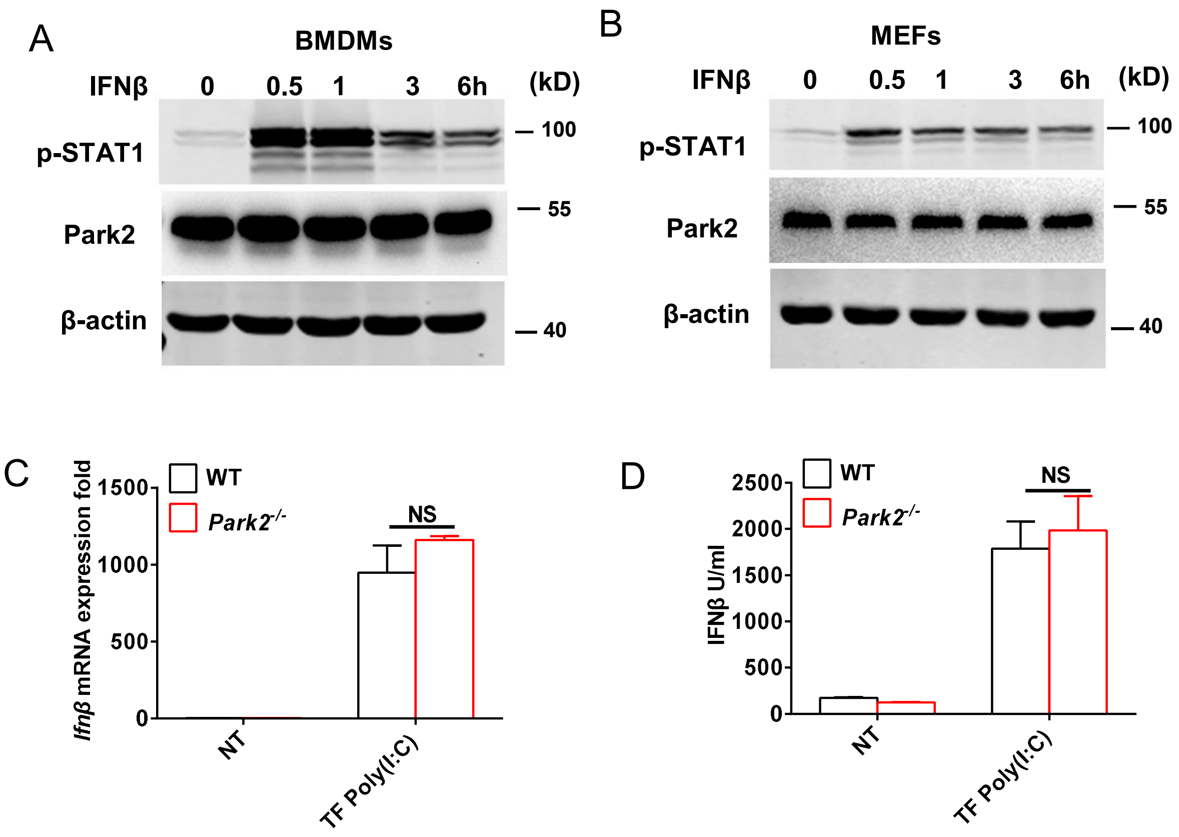

Figure S8. The independent role of type I IFNs in regulating Parkin expression in response to virus infection, and IFN production in response to RLR non replicative IFN inducer, Related to Figure 1.

(A) Immunoblot analysis of phosphorylated (p-) STAT1, Park2 and β-actin (loading control) in WT BMDMs treated with IFNβ (500U/ml) for 0,0.5, 1, 3 and 6h.

(B) Immunoblot analysis of phosphorylated (p-) STAT1, Park2 and β-actin (loading control) in WT MEFs treated with IFNβ (500U/ml)for 0,0.5, 1, 3 and 6h.

(C) RT-qPCR analysis of *Ifnb* mRNA expression of WT and *Park2*$^{-/-}$ MEFs transfected with Poly(I:C) (10ug/ml) for 6h. NT, No treatment. TF: Transfect.

(D) ELISA analysis of IFN-β protein expression of WT and *Park2*$^{-/-}$ MEFs transfected with Poly(I:C) (10ug/ml) for 18h. NT, No treatment. TF: Transfect.

Data are pooled from three independent experiments. Error bars show means ± SEM. NS, not significant. Two-way ANOVA with Sidak's multiple comparisons test.
